# Cultural variation in age perceptions and developmental transitions

**DOI:** 10.3389/frsps.2023.1283643

**Published:** 2024-01-04

**Authors:** Michelle E. Vargas, Alejandro Carrillo, Hannah L. Giasson, William J. Chopik

**Affiliations:** 1 Department of Psychology, Northeastern Illinois University, Chicago, IL, United States; 2 Department of Psychology, Michigan State University, East Lansing, MI, United States; 3 Edson College of Nursing and Health Innovation, Arizona State University, Phoenix, AZ, United States

**Keywords:** age perceptions, developmental transitions, project implicit, age differences, age-group dissociation, collectivism

## Abstract

As people age, they increasingly report feeling younger than their actual age and “push off” when they think older adulthood starts, presumably to create and maintain psychological distance from the stigma of being an older adult. However, to date, such age perceptions and attitudes have mostly been studied in Western cultures (e.g., the United States and Europe). However, cultures vary in their perception of older adulthood and aging, suggesting that the extent to which people engage in these distancing processes might differ across cultures. In the current study, we examined age differences in age perceptions and perceived developmental transitions (e.g., when does someone move from middle age to older adulthood?) in 13 countries with over one million people total. We found that age-group distancing was present in each country but that this pattern was less pronounced in South Korea. Results are discussed in the context of cross-cultural variation in aging attitudes and the mechanisms that give rise to variation in age perceptions.

## Introduction

As people age, they increasingly report feeling younger than their actual age and “push off” when they think older adulthood starts, presumably—among many reasons—to create and maintain psychological distance from the stigma of being an older adult (e.g., Kotter-Grühn et al., 2009; Weiss and Lang, 2012; Ayalon et al., 2014; Chopik et al., 2018; Weiss and Kornadt, 2018; Jurek, 2022). However, cultures vary in their perception of older adulthood and aging (North and Fiske, 2015a,b). As a result, the extent to which people engage in these distancing processes might differ across cultures and could be a way to infer how those cultures think about aging (which has implications for group dynamics and policy). In the current study, we examined age differences in age perceptions and perceived developmental transitions in 13 countries among over one million people.

### Age perceptions and the age-group dissociation effect

As people age, they increasingly report wanting to be younger (if they could choose any age), report that other people think they are younger, and also “push off” when middle age starts (Chopik et al., 2018). Changes in these perceptions can be partially explained by the *age-group dissociation effect* (Weiss and Lang, 2012). Put simply, for many people, older adulthood is a time and status in life that is rife with stigma, including negative perceptions about competence, appearance, and utility (Cuddy and Fiske, 2002). As a result, people employ strategies to “distance” themselves from this status as they do with many other statuses and states (Tajfel, 1969; Ellemers et al., 1997; Mussweiler et al., 2000; Cesario et al., 2010). Both experimental and correlational evidence suggest that, when confronted with negative stereotypes about older adults, people will identify with younger adults (by saying they feel younger and claiming that other people think they are younger), limit social interactions with older adults, and divert their attention away from older adults in their environment (Weiss and Freund, 2012; Weiss and Lang, 2012; Weiss et al., 2013; Weiss and Kornadt, 2018). Another way that people distance themselves from the idea that they are old is through other motivational evaluations, like altering the definition of what an older adult is and when older adulthood starts. Similar to the ways that people identify with younger people as they get older, they also continuously push older adulthood into the future as they age (Kuper and Marmot, 2003; Taylor et al., 2009). For example, young adults (aged 18–29) think that older adulthood starts at age 60, but adults over the age of 65 think that older adulthood does not start until after age 74 (Taylor et al., 2009).

### Cultural variation in age perceptions

By assessing cultural differences in age perceptions, we can somewhat infer how those cultures feel about the aging process, without asking them explicitly how they feel about older adults directly. This is an important consideration because cultures differ in how they think about older adults and their roles in society, which may have downstream consequences for how people think about themselves aging. For example, variation in age attitudes and age perceptions are linked to how well individuals age (both mentally and physically; Levy and Myers, 2004; Levy et al., 2009), how much regions spend on age-related costs and expenditures (Giasson and Chopik, 2020; Levy et al., 2020), and people’s support for age-friendly policies, even when they interfere with their own self-interest (Levy and Schlesinger, 2005). Given the link between age attitudes and perceptions and outcomes for both individuals and societies, examining cultural variation in these characteristics has been suggested as one possible avenue for explaining health and wellbeing disparities for older adults across cultures (Levy et al., 2016; Giasson and Chopik, 2020; Ayalon and Cohn-Schwartz, 2022). One way of evaluating how people think about aging and older adulthood is to examine how they think about their own age and when they think various developmental transitions occur across the lifespan.

Why would age perceptions vary across cultures? People living in East Asian and other collectivist cultures are more likely to place older adults in a respected and venerated position, likely because of their familism and more integrated family networks (Sung, 2001). This reflects a broader context of people from collectivistic cultures holding more positive attitudes about older adults (Boduroglu et al., 2006; Xiao et al., 2013; Vauclair et al., 2017; Tan and Barber, 2018). In a large English-speaking sample of 68 countries, Ackerman and Chopik (2021) found that people living in more collectivistic cultures reported less age bias and greater feelings of warmth toward older adults. However, there is also work suggesting that many of these same cultures might show more *negative* attitudes toward older adults because of the strain a rapidly aging population places on society (North and Fiske, 2015a,b, 2016; North, 2022). Supporting this possibility, a series of studies show that attitudes toward older adults are more negative in the Eastern cultures, particularly those with dramatically aging populations (Löckenhoff et al., 2009; Huang, 2013).

To date, most of this work has focused on subjective age (i.e., how old people feel), with only a handful of cross-cultural studies examining developmental transitions, and mostly only focused on European countries (Ayalon et al., 2014; Augustyński and Jurek, 2021; Jurek, 2022). The few studies that have been conducted find that, across cultures, older adults report a younger subjective age (e.g., Van Auken et al., 2006; Hess et al., 2017; O’Brien et al., 2017; Liang, 2020; Ackerman and Chopik, 2021). Hess and colleagues (2017) note that some countries have age considerations tied into societal structures (e.g., mandatory retirement ages), so they may have more formalized perceptions of older adults (e.g., older adults cannot or should not work beyond a certain point). One study has shown that the age differences in subjective age are present in most cultures but do not necessarily differ in magnitude according to often-studied cultural dimensions (Ackerman and Chopik, 2021). Yet other studies have shown that not all older adult samples report a younger subjective age (Macia et al., 2012; Schönstein et al., 2021, 2023). Finally, a recent meta-analysis similarly found heterogeneity in lifespan differences in subjective age across countries, and these effects might be stronger in Western countries (Pinquart and Wahl, 2021).

The inconsistent findings from previous work leave ambiguity about whether lifespan differences in age perceptions and developmental transitions exist in different cultures and if they are of the same magnitude (Löckenhoff et al., 2009; Kornadt et al., 2022). Given the ambiguity of previous research, we approached cultural variation in these processes as an open question.

## Method

### Participants and procedure

Participants were 1,007,956 individuals who took part in the Project Implicit International Project, an effort to collect information on the Implicit Association Test across multiple cultures and languages (https://osf.io/kaqi5/; Charlesworth et al., 2023). The Project Implicit International Project constitutes a partnership of 34 individual websites hosting implicit bias-related tests and instruments in a variety of languages. Measures were harmonized across sites and translated into the most common local language (although multiple languages per country were available, such as French and German for Switzerland). The current study reports on new questionnaire information from 12 countries–Australia (*N* = 21,754), Belgium (*N* = 2,262), Brazil (*N* = 6,158), Canada (*N* = 28,088), China (*N* = 10,066), France (*N* = 17,343), Germany (*N* = 19,234), Korea (*N* = 8,057), Netherlands (*N* = 9,970), Spain (*N* = 9,097), Sweden (*N* = 11,338), and the United Kingdom (*N* = 45,965). Data from a 13th country (the United States; *N* = 818,624) was added to allow comparisons to previous research (Chopik et al., 2018).

Sample characteristics ranged across countries (*M*_*age*_ ranged from 24.07 [China] to 32.87 [UK]; 51.1% [Brazil] to 73.1% [Korea] women; see Table 1 for full descriptives across cultures; total sample ranged in age from 10 to 89; *M*_age_ = 27.45, *SD* = 12.45; 67.1% women). Data from each country were collected over different time intervals (many of which were overlapping). An additional 865 people were excluded for providing ages younger than age 10.^1^

The Michigan State Institutional Review Board considered this research exempt from ethical oversight as it did not constitute human subjects research (IRB# 17–1113). Data and syntax for this report can be found at https://osf.io/h84pd/.

### Evaluative perceptions about aging

Participants received four open-ended questions asking which age they would choose to be (“If you could choose, what age would you be?”; hereafter *age choice* in all tables), what age they felt like (“How old do you feel?”; *subjective age*), what age they hope to live until (“To what age do you hope to live?”; *hope to live*), and how old other people think they are (“On average, how old do other people think you are?”; *perceived age*). Descriptives for these questions across countries can be found in Table 1.

For some countries (particularly US, Canada, and UK), participants received a subsample of questions (either the age perception or developmental transition questions). Degrees of freedom for each analysis can be found in the notes for Supplementary Tables 1–8.

### Age estimates for developmental transitions

Participants also received four open-ended questions asking the age at which four different developmental transitions occurred. The four transitions were from childhood to young adulthood (“A person moves from being a child to being a young adult at what age?”), from young adulthood to adulthood (“A person moves from being a young adult to being an adult at what age?”), from adulthood to middle-aged (“A person moves from being an adult to middle-aged at what age”), and from middle-aged to older adulthood (“A person moves from being middle-aged to being old at what age?”).

### Statistical analysis

Within each country, we conducted regression analyses predicting each outcome (i.e., four age perceptions and four developmental transitions) from age, age^2^, and gender. Age was centered prior to computing these higher-order terms in order to reduce multi-collinearity. Gender was included as a control variable in each model given research on gendered perceptions of what is considered an older adult (Zepelin et al., 1987; Seccombe and Ishii-Kuntz, 1991; McConatha et al., 2003).^2^ Following a variant of previous research (Choi et al., 2014), we subtracted each age perception variable (i.e., subjective age, perceived age, and chosen age) from participants’ chronological age, such that increasingly *higher values* suggest *greater* age distancing (i.e., by saying people felt younger, are perceived to be younger, and would choose a younger age than their current chronological age).^3^

To draw comparisons between countries, we primarily focus on the linear effect of age as provides an unambiguous test of age-group dissociation effects (i.e., the larger the association between age and the perceived transition to older adulthood suggests greater age-group dissociation). We primarily focus on effect sizes, but full regression results can be found in Supplementary Tables 1–8.

## Results

As seen in Table 1, there was variation in the age perceptions and developmental transitions. Given the large sample sizes, traditional ANOVA tests revealed mostly significant differences in pairwise comparisons between countries.

The largest cross-cultural difference was for the transition from adulthood to middle age (*η*^2^ = 0.125) with estimates ranging from as low as 38.75 (Germany) to 53.04 (Spain). The next largest difference was for the transition from childhood to young adulthood (*η*^2^ = 0.059) with estimates ranging from 12.70 (China) to 17.30 (France). Modest effect sizes were also seen for the middle-age to older adult transition (57.57 in China and 71.53 in Belgium; *η*^2^ = 0.045), the young adult to adult transition (18.94 in China and 24.94 in France; *η*^2^ = 0.039), and perceived age ([how old people think you are] 23.94 in South Korea and 31.43 in the United Kingdom; *η*^2^ = 0.038). China tended to give the lowest ages for developmental transitions, but there was otherwise no clear pattern. Other cross-cultural differences in the other outcomes were relatively small (remaining *η*^2^s ranged from 0.015 to 0.017).

### Age perceptions

**FIGURE 1:**
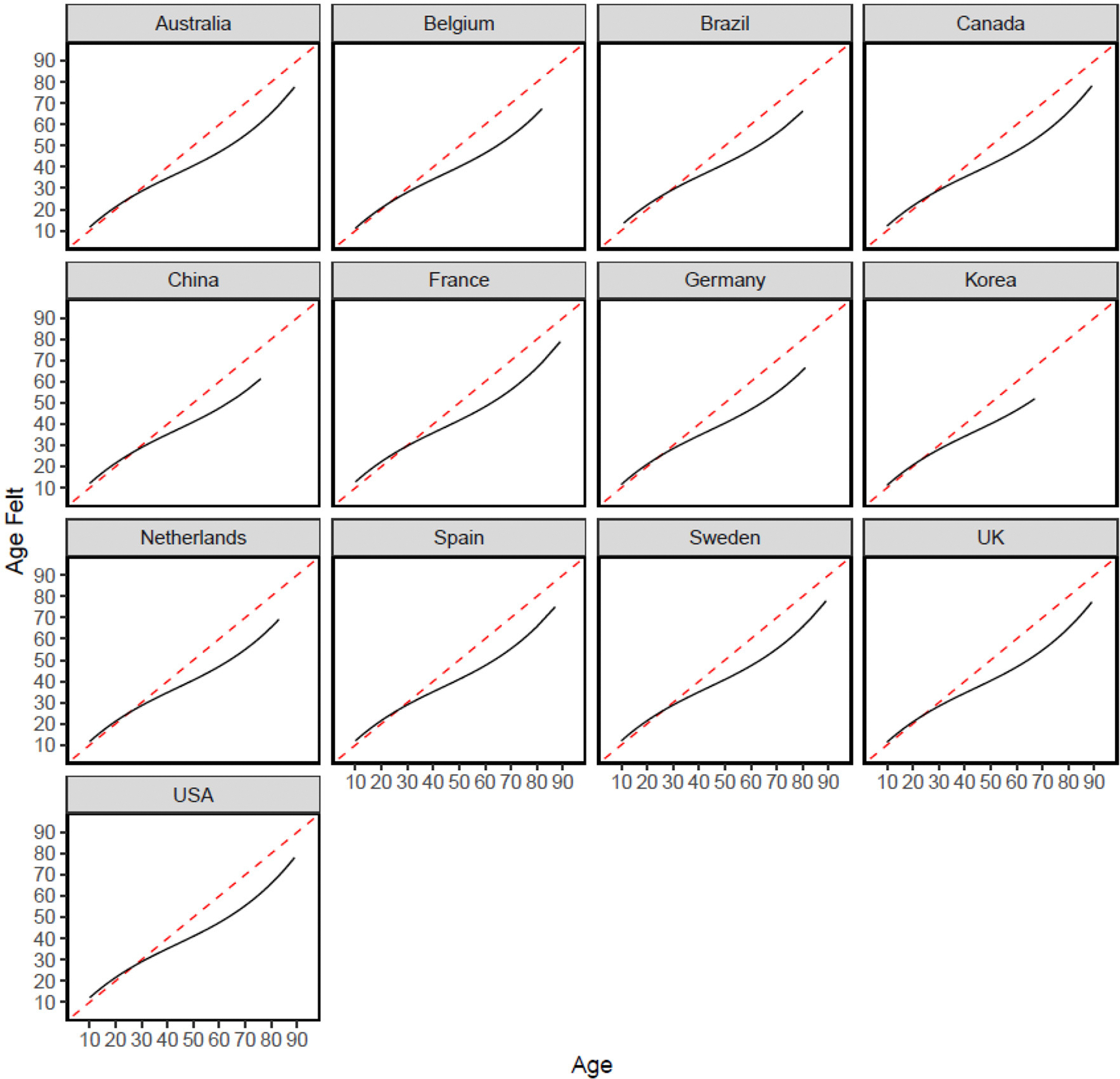
Subjective age effects across 13 countries. Model-implied regression line (for age) is plotted. Red dashed line represents the identity line (i.e., if people felt their chronological age across the lifespan).

Replicating previous research, when people are younger, they reported feeling the same age as their biological age (see Figure 1).^4^However, people in their 20s and 30s begin reporting feeling younger ages than their biological age, and this difference (between their felt and actual age) was stable among people in their 50s and 60s.

The same effect can be seen for the age other people think they are (Supplementary Figure 1) and the age people choose to be (Supplementary Figure 2); however, the effect was more dramatic for the age people choose to be (i.e., regardless of age, people tended to choose ages 50 and younger). People hope to live to high ages, and this did not differ much by age (Supplementary Figure 3).

**FIGURE 2:**
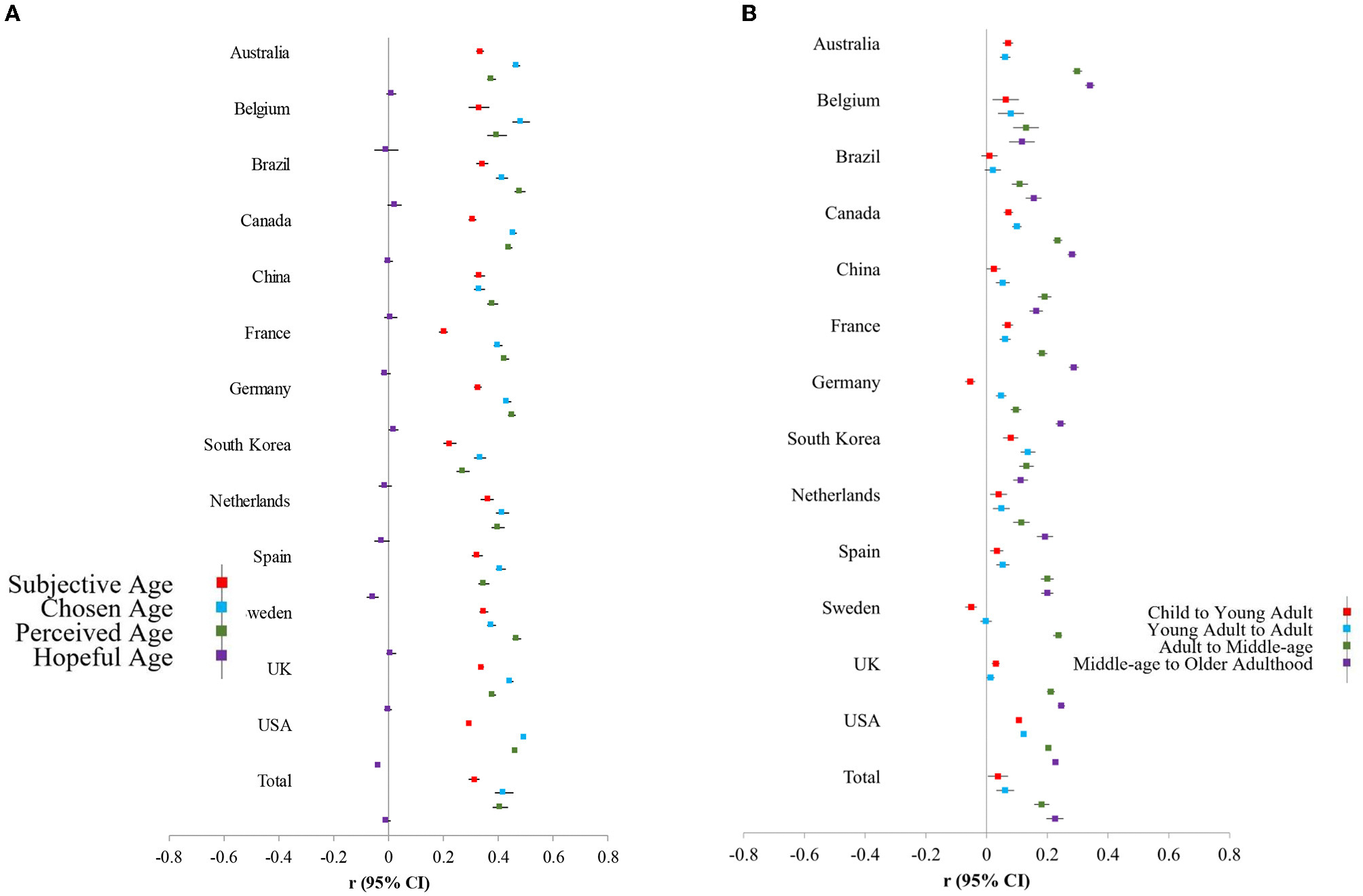
Age differences in age perceptions **(A)** and developmental transitions **(B)** across countries. Effects of age from regressions were converted to r effect sizes to represent the magnitude of age-group dissociation across cultures.

Figure 2A summarizes cross-cultural variation in the magnitude of the associations between age and subjective age (red marker), chosen age (blue), perceived age (green), and age that they hope to live until (purple); an overall effect size from a random-effects meta-analysis on each variable is also provided. In particular, larger effect sizes suggest a larger correlation between age and age-group distancing.

For subjective age (red), the smallest effect sizes were seen for France and South Korea; the remaining effect sizes were comparable across cultures. This suggests that age-group dissociation is lower in France and South Korea with respect to subjective age. For chosen age (blue), the smallest effect sizes were again found for South Korea and China, and the largest effect sizes were seen among the U.S. and Belgium. For perceived age (green), the smallest effect size was South Korea, and the largest effect size was Brazil. Collectively, there were not many straightforward patterns to the heterogeneity across countries (e.g., collectivistic cultures were often comparable to individualistic cultures). However, across all age perception measures, South Korea reported the lowest age-group dissociation.

Consistent with the interpretation above, the age people would like to live until was mostly unrelated to age (purple).

### Developmental transitions

**FIGURE 3:**
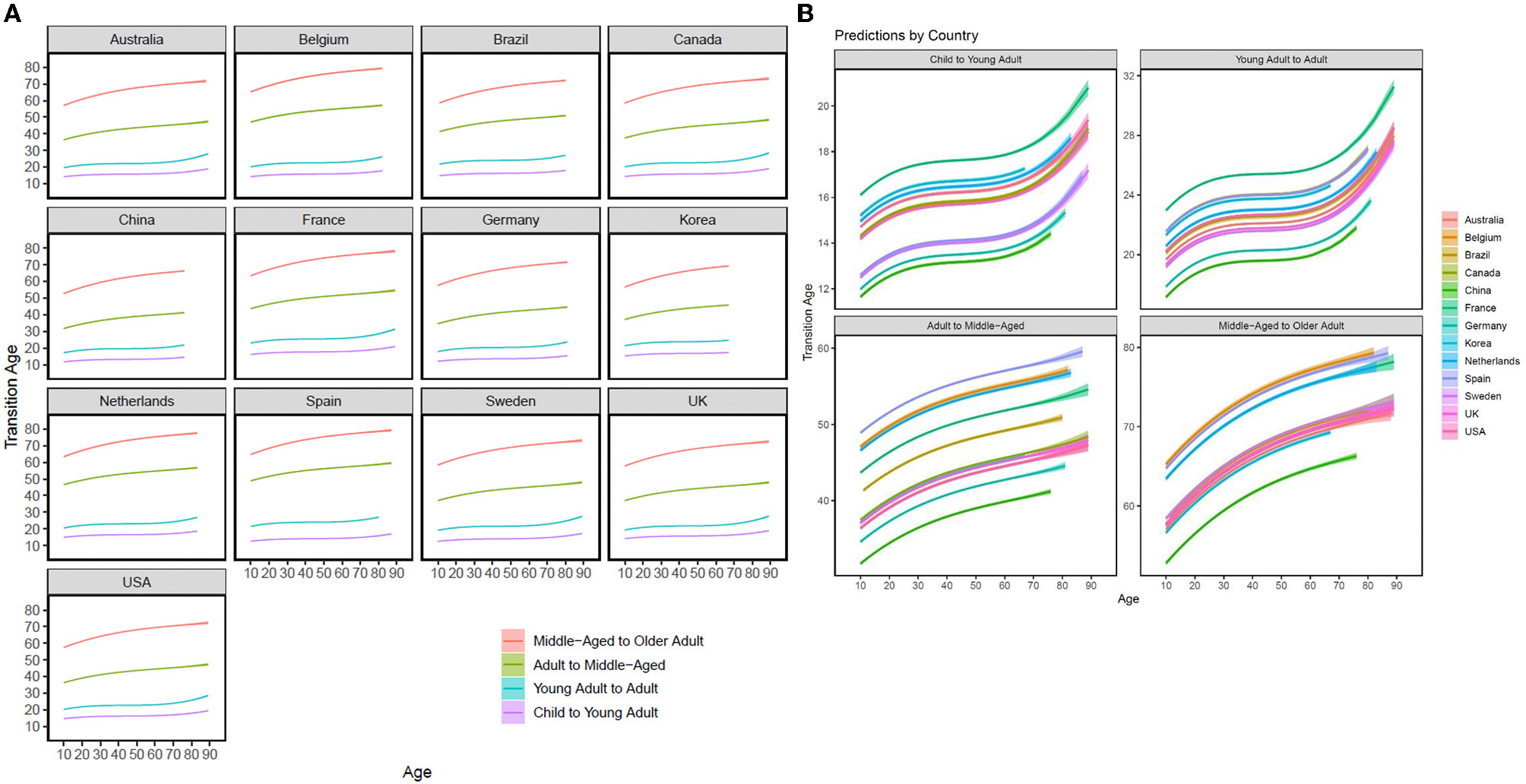
Perceived developmental transitions across 13 countries by country (A) and by outcome (B). Model-implied regression lines (for age) are plotted for each transition. Confidence intervals are also provided. See Supplementary material for zoomed-out (B).

As seen in Figures 3A, B, in every culture, age differences for the child-to-young-adult (purple) and young-adult-to-adult (blue) transitions were relatively flat as previous theory would suggest that people do not “push off” the transitions around young adulthood (as they are not stigmatized). There were stark age differences in the adult-to-middle-age and middle-age-to-older-adulthood transition. Middle-aged and older adults tended to report that middle-age and older adulthood started at later ages (than younger adults). Thus, some degree of age group dissociation in terms of developmental transitions is present in every country examined.

Figure 2B summarizes cross-cultural variation in the magnitude of associations between age and each transition. Consistent with Figure 3 and past research, there is a “fanning” pattern in which younger transitions (i.e., red and blue markers) are closer to zero, and older transitions (green and purple markers) have larger magnitudes. Exceptions to this fanning pattern were South Korea and Belgium, who demonstrated comparable age differences in each of the developmental transitions. The countries with the largest effect sizes (i.e., the greatest age-group dissociation) were Australia, Sweden, Canada, and France.

## Discussion

We replicated the effect seen in previous research that as people age they identify with younger age groups (Kuper and Marmot, 2003; Rubin and Berntsen, 2006; Taylor et al., 2009; Augustyński and Jurek, 2021; Jurek, 2022). We found these patterns in all 13 countries we had access to in the current report. When examining age differences in these processes, we found modest meta-analytic effect sizes for subjective age (*r* = 0.313), chosen age (*r* = 0.422), perceived age (*r* = 0.409), the transition from adulthood to middle age (*r* = 0.182), and the transition from middle age to older adulthood (*r* = 0.226). We did not find as robust associations for other developmental transitions that are not stigmatized (*r* = 0.038 for childhood to young adulthood and *r* = 0.061 for young adulthood to adulthood) or the age that people hoped they would live to (*r* =−0.005) which should be unrelated to age-group dissociation processes.

Findings aligned with studies conducted among North American and European participants. However, we also examined how these associations varied across cultures. In the current study, few countries emerged as having reliably larger or smaller age-group dissociation tendencies (i.e., defined as the magnitude of age differences in age perceptions and developmental transitions). South Korea, often considered to be a collectivistic country, had the smallest effect size across a variety of outcomes. Beyond this consistent result, other countries also had smaller age group dissociation tendencies depending on the outcome, including France, Belgium, and Brazil—countries that vary along the individualism/collectivism dimension. The variation is also not readily attributable to countries that have struggled with population aging.

Why were there mixed results regarding cultural variation in age perceptions? Previous research was mixed—some research would suggest that we would see smaller age group dissociation in collectivistic cultures (Ackerman and Chopik, 2021), but others suggested that we might have seen larger effect sizes in some of these same cultures given the strain introduced by a rapidly aging population (North and Fiske, 2016). We actually found a third pattern—that most associations were similar in magnitude and there was a large degree of consistency across cultures. For example, most age differences in the middle-age-to-older-adulthood transition ranged from around *r* = 0.20 to *r* = 0.30. One possibility for the homogeneous results across countries is that people from each of these countries have at least some reservations about becoming older and view aging in a negative light. In other words, past research’s characterization of collectivistic cultures as being more positive about older adults may have obscured the fact that those same cultures may still feel a bit negatively about aging (even if they are more positive than those in individualistic cultures). Indeed, a careful examination of past work finds that some of these processes are present in most cultures even if they show some relative variation when comparing countries (Ackerman and Chopik, 2021). Why some countries bucked this trend (e.g., South Korea) is a bit of a mystery. One potential area of future research is to conduct a more proximal analysis of the particular age-related concerns and aging ecosystem of individual countries. It could be that South Korea and other countries might have extenuating circumstances that are specific to their society which might explain why they engage in less age-group distancing. Analyzing specific cultures and their local attitudes might be a more fruitful avenue than analyzing variation in age perceptions at such a coarse level as done here.

### Limitations and future directions

Although this study was among one of the few cross-cultural investigations of age perceptions and perceived developmental transitions (Augustyński and Jurek, 2021; Jurek, 2022), we had too few countries to formally model explanatory predictors of variation in these effects (e.g., characteristics like individualism/collectivism). We chose the countries based on the data publicly available to us rather than a more systematic sampling of countries, which was also a limitation. As more cross-cultural data collections include these measures, more comprehensive cross-cultural examinations will become feasible, possibly in aggregation with our and others’ data (Augustyński and Jurek, 2021; Jurek, 2022).

The cross-sectional nature of the data prevents causal statements about developmental changes in age perceptions and transitions over the life course. Although longitudinal studies on these concepts have been done (Markides and Boldt, 1983; Van Auken et al., 2006; Ward, 2013; Bergland et al., 2014; Ayalon et al., 2016), doing so in the context of cross-cultural comparisons to examine the rate of these changes over time in several different cultures has not. Sensitivity to the many different contexts that people find themselves in—both in time and space—would be appropriate to examine in terms of how these characteristics affect age attitudes (Giasson and Chopik, 2020; Rupprecht et al., 2022; Wettstein et al., 2023).

Finally, we did not directly test any of the mechanisms explaining why people might shift their age perceptions, although there are many ideas that researchers have generated (Kotter-Grühn et al., 2016). Where exactly do these age perceptions and developmental transitions come from? What exactly is subjective age? Where and when do people form ideas about when older adulthood starts? There is at least some consensus that a construct like subjective age partially reflects people’s current mental and physical health, how they are adapting to age-related losses, and their attitudes toward their own and others’ aging (Levy, 2003; Kotter-Grühn et al., 2016). Others think age perceptions might reflect a “denial” of death such that reporting a younger age serves a particular function to preserve one’s sense of self (Bultena and Powers, 1978). Given subjective age’s late life declines and links with mortality (Kotter-Grühn et al., 2009), it could also be the case that subjective age might at least partially capture people’s assessments of their longevity, too (i.e., how long they think they’ll live). Future research can more deliberately test these competing ideas against each other in the context of a longitudinal study.

## Conclusion

In each country, some degree of age-group dissociation was found in which people reported feeling, choosing to ideally be, and being perceived as a younger age than their chronological age. People from each culture also “pushed off” when middle age and older adulthood started, although these effects were more muted in South Korea. Given the important outcomes linked to people’s age perceptions (Ayalon et al., 2014; Mejía et al., 2020), it will be important to quantify sources of variation in these perceptions given that concerns about aging have persisted across generations and have not improved despite improvements in other areas of life for older adults (Wahl et al., 2022).

## Supplementary Material

Supplementary material

The Supplementary Material for this article can be found online at: https://www.frontiersin.org/articles/10.3389/frsps.2023.1283643/full#supplementary-material

## Figures and Tables

**TABLE 1:** Descriptives and study variables across countries.

Country	*N*	*Age* (*M*)	*Age* (*SD*)	Gender (% women)	If you could choose, what age would you be?	How old do you feel?	On average, how old do other people think you are?	To what age do you hope to live?	A person moves from being a child to being a young adult at what age?	A person moves from being a young adult to being an adult at what age?	A person moves from being an adult to middle-aged at what age?	A person moves from being middle-aged to being old at what age?	Data collection period
Australia	21,754	30.02	13.57	60.4	26.06	28.56	29.01	87.31	15.38	21.61	40.80	63.32	2004–2016
Belgium	2,262	31.32	14.45	57.7	26.05	28.51	29.65	86.25	15.49	22.14	51.46	71.53	2009–2017
Brazil	6,158	29.34	10.86	51.1	24.70	28.51	26.46	84.96	15.89	23.56	45.34	64.22	2008–2017
Canada	28,088	29.34	13.30	63.8	25.47	27.48	26.87	88.35	15.46	22.01	41.44	64.12	2004–2016
China	10,066	24.07	6.52	55.1	21.65	24.52	22.90	84.13	12.70	18.94	35.17	57.57	2006–2017
France	17,343	30.22	12.78	61.7	26.40	29.12	28.30	86.88	17.30	24.94	47.96	69.57	2005–2016
Germany	19,234	29.32	11.94	60.4	25.61	27.28	27.04	87.19	13.14	19.76	38.75	63.50	2006–2016
Korea	8,057	25.13	7.57	73.1	21.04	23.94	23.54	80.67	16.24	23.06	40.67	61.60	2006–2017
Netherlands	9,970	31.57	14.04	63.9	26.50	28.47	29.27	87.37	16.12	22.49	50.96	69.65	2006–2017
Spain	9,097	29.07	13.43	66.2	24.98	27.93	29.34	85.52	13.76	23.47	53.04	70.60	2008–2017
Sweden	11,338	31.35	13.94	64.5	27.89	29.05	29.30	92.40	13.71	21.17	41.49	64.77	2007–2017
UK	45,965	32.87	12.99	53.1	27.22	30.36	31.43	87.18	15.46	21.46	41.98	64.87	2004–2016
USA	81,8624	26.83	12.27	68.7	24.08	25.78	24.64	90.00	15.75	22.01	40.01	62.69	2004–2016
Total sample	1,007,956	27.45	12.45	67.1	24.56	26.51	25.71	89.09	15.54	21.97	40.90	63.37	2004–2017

## Data Availability

Publicly available datasets were analyzed in this study. This data can be found here: https://osf.io/h84pd/.

## Footnotes

1 Some of the countries (e.g., China and South Korea) had very few participants over the age of 70 (this is reflected in the figures as we did not plot small sample sizes). As a conservative robustness check, we re-ran our analyses excluding everyone over the age of 70 (among people for whom the age-group dissociation is likely largest). The results remained largely unchanged as evidenced by the preserved rank-order of the countries by effect size (*r* = 0.98) and the small changes in the standardized effects with these new analyses (Δ*βs* < |0.008|). Because the effect sizes for most of the variables are relatively large and restricting the sample to only contain the most representative ages yielded similar effects, the smaller samples at advanced ages likely did not unduly influence the results.

2 In examining whether gender moderated the effects of age, we found mostly mixed results for most countries and most outcomes (after applying a Bonferroni correction): subjective age (moderation only present in 3 of 13 countries), perceived age (3 countries), hope to live (1 country), child-to-young adult transition (1 country), young adult-to-adult transition (1 country), adult-to-middle-age transition (1 country), and middle-age-to-older-adult transition (2 countries). The one exception was the age choice outcome (11 of 13 countries). Specifically, among Americans for example, decomposing this interaction revealed that the slope of age was larger for women (*β* = 0.67, *p* < 0.001) compared to men (*β* = 0.53, *p* < 0.001), such that older women reported a higher age choice than men, which is consistent with the main effect of gender that is occasionally seen across models (see Supplementary material).

3 An alternative approach is to model these associations in the context of multi-level modeling in which participants are nested within countries. However, given the small number of Level-2 units (i.e., 13; see McNeish and Stapleton, 2016 for a discussion of small cluster numbers in the context of multi-level modeling) and the homogeneous sampling of countries (e.g., most were from Europe), we elected to run the analyses separately within each country and examine variation in the effect sizes in the context of a meta-analysis.

4 In most cases, the youngest people in the sample reported feeling *older* than their current chronological age, consistent with a cross-over effect seen in previous research (Galambos et al., 2005). This cross-over effect happened around age 27–29 for people from Australia, Belgium, Brazil, Canada, and the United States. The cross-over was a little younger (25–26) for people from China, Spain, Sweden, and the United Kingdom (most consistent with Galambos et al., 2005). Finally, this cross-over was youngest among people from South Korea (20–21) and those from France, Germany, and the Netherlands (22–23).
