# Supplementary material for: Cultural variation in age perceptions and developmental transitions
Source: Front Soc Psychol. Author manuscript; Available in PMC 2026 Jan 13. (PMC12794726; doi:10.3389/frsps.2023.1283643)

| Supplementary Table 1. Results for subjective age | | | | | | | | | | | | | | |  |
| --- | --- | --- | --- | --- | --- | --- | --- | --- | --- | --- | --- | --- | --- | --- | --- |
| Australia | *b* | | *SE* | | *β* | | *t* | | *p* | | 95% LB | | 95% UB | |  |
| Age | 0.304 | | 0.008 | | 0.484 | | 40.236 | | < 0.001 | | 0.289 | | 0.319 | |  |
| Age^2^ | 0.002 | | < 0.001 | | 0.069 | | 5.764 | | < 0.001 | | 0.001 | | 0.003 | |  |
| Gender | 0.316 | | 0.068 | | 0.035 | | 4.660 | | < 0.001 | | 0.183 | | 0.449 | |  |
| Note. *F*(3,12830) = 1726.126, *p* < .001. *R*^2^ = .288 | | | | | | | | |  | |  | |  | |  |
|  |  | |  | |  | |  | |  | |  | |  | |  |
| Belgium | *b* | | *SE* | | *β* | | *t* | | *p* | | 95% LB | | 95% UB | |  |
| Age | 0.287 | | 0.018 | | 0.534 | | 16.048 | | < 0.001 | | 0.252 | | 0.322 | |  |
| Age^2^ | < 0.001 | | 0.001 | | 0.019 | | 0.561 | | 0.575 | | -0.001 | | 0.002 | |  |
| Gender | 0.202 | | 0.148 | | 0.026 | | 1.362 | | 0.173 | | -0.089 | | 0.492 | |  |
| Note. *F*(3,2073) = 292.438, *p* < .001. *R*^2^ = .297 | | | | | | | | | | | | | | |  |
|  |  | |  | |  | |  | |  | |  | |  | |  |
| Brazil | *b* | | *SE* | | *β* | | *t* | | *p* | | 95% LB | | 95% UB | |  |
| Age | 0.367 | | 0.014 | | 0.470 | | 26.918 | | < 0.001 | | 0.341 | | 0.394 | |  |
| Age^2^ | < 0.001 | | 0.001 | | -0.003 | | -0.194 | | 0.846 | | -0.001 | | 0.001 | |  |
| Gender | -0.009 | | 0.102 | | -0.001 | | -0.086 | | 0.931 | | -0.209 | | 0.191 | |  |
| Note. *F*(3,5438) = 506.245, *p* < .001. *R*^2^ = .218 | | | | | | | | | | | | | | |  |
|  |  | |  | |  | |  | |  | |  | |  | |  |
| Canada | *b* | | *SE* | | *β* | | *t* | | *p* | | 95% LB | | 95% UB | |  |
| Age | 0.305 | | 0.008 | | 0.456 | | 39.859 | | < 0.001 | | 0.290 | | 0.320 | |  |
| Age^2^ | 0.001 | | < 0.001 | | 0.043 | | 3.772 | | < 0.001 | | 0.001 | | 0.002 | |  |
| Gender | 0.312 | | 0.066 | | 0.033 | | 4.703 | | < 0.001 | | 0.182 | | 0.442 | |  |
| Note. *F*(3,15455) = 1640.202, *p* < .001. *R*^2^ = .241 | | | | | | | | |  | |  | |  | |  |
|  |  | |  | |  | |  | |  | |  | |  | |  |
| China | *b* | | *SE* | | *β* | | *t* | | *p* | | 95% LB | | 95% UB | |  |
| Age | 0.393 | | 0.013 | | 0.357 | | 30.073 | | < 0.001 | | 0.368 | | 0.419 | |  |
| Age^2^ | -0.003 | | 0.001 | | -0.029 | | -2.471 | | 0.013 | | -0.005 | | -0.001 | |  |
| Gender | 0.546 | | 0.081 | | 0.074 | | 6.702 | | < 0.001 | | 0.386 | | 0.705 | |  |
| Note. *F*(3,7299) = 333.471, *p* < .001. *R*^2^ = .120 | | | | | | | | | | | | | | |  |
|  |  | |  | |  | |  | |  | |  | |  | |  |
| France | *b* | | *SE* | | *β* | | *t* | | *p* | | 95% LB | | 95% UB | |  |
| Age | 0.099 | | 0.004 | | 0.317 | | 22.259 | | < 0.001 | | 0.090 | | 0.108 | |  |
| Age^2^ | 0.001 | | < 0.001 | | 0.063 | | 4.395 | | < 0.001 | | 0.001 | | 0.001 | |  |
| Gender | -0.008 | | 0.036 | | -0.002 | | -0.232 | | 0.817 | | -0.079 | | 0.062 | |  |
| Note. *F*(3,11743) = 618.133, *p* < .001. *R*^2^ = .136 | | | | | | | | | | | | | | |  |
|  |  | |  | |  | |  | |  | |  | |  | |  |
| Germany | *b* | | *SE* | | *β* | | *t* | | *p* | | 95% LB | | 95% UB | |  |
| Age | 0.281 | | 0.007 | | 0.499 | | 39.326 | | < 0.001 | | 0.267 | | 0.295 | |  |
| Age^2^ | -0.002 | | < 0.001 | | -0.061 | | -4.833 | | < 0.001 | | -0.002 | | -0.001 | |  |
| Gender | 0.122 | | 0.055 | | 0.018 | | 2.225 | | 0.026 | | 0.015 | | 0.230 | |  |
| Note. *F*(3,12946) = 1102.685, *p* < .001. *R*^2^ = .203 | | | | | | | | |  | |  | |  | |  |
|  |  | |  | |  | |  | |  | |  | |  | |  |
| Korea | *b* | | *SE* | | *β* | | *t* | | *p* | | 95% LB | | 95% UB | |  |
| Age | 0.227 | | 0.013 | | 0.288 | | 18.045 | | < 0.001 | | 0.202 | | 0.251 | |  |
| Age^2^ | -0.003 | | 0.001 | | -0.051 | | -3.265 | | 0.001 | | -0.004 | | -0.001 | |  |
| Gender | 0.233 | | 0.084 | | 0.036 | | 2.784 | | 0.005 | | 0.069 | | 0.398 | |  |
| Note. *F*(3,6167) = 140.492, *p* < .001. *R*^2^ = .064 | | | | | | | | | | | | | | |  |
|  |  | |  | |  | |  | |  | |  | |  | |  |
| Netherlands | *b* | | *SE* | | *β* | | *t* | | *p* | | 95% LB | | 95% UB | |  |
| Age | 0.293 | | 0.011 | | 0.587 | | 27.358 | | < 0.001 | | 0.272 | | 0.314 | |  |
| Age^2^ | -0.001 | | < 0.001 | | -0.032 | | -1.489 | | 0.137 | | -0.002 | | 0.000 | |  |
| Gender | 0.083 | | 0.088 | | 0.011 | | 0.943 | | 0.346 | | -0.090 | | 0.256 | |  |
| Note. *F*(3,4999) = 757.029, *p* < .001. *R*^2^ = .312 | | | | | | | | | | | | | | |  |
|  |  | |  | |  | |  | |  | |  | |  | |  |
| Spain | *b* | | *SE* | | *β* | | *t* | | *p* | | 95% LB | | 95% UB | |  |
| Age | 0.258 | | 0.008 | | 0.565 | | 31.134 | | < 0.001 | | 0.242 | | 0.274 | |  |
| Age^2^ | -0.001 | | < 0.001 | | -0.078 | | -4.328 | | < 0.001 | | -0.002 | | -0.001 | |  |
| Gender | 0.076 | | 0.063 | | 0.012 | | 1.220 | | 0.223 | | -0.046 | | 0.199 | |  |
| Note. *F*(3,8250) = 913.543, *p* < .001. *R*^2^ = .249 | | | | | | | | | | | | | | |  |
|  |  | |  | |  | |  | |  | |  | |  | |  |
| Sweden | *b* | | *SE* | | *β* | | *t* | | *p* | | 95% LB | | 95% UB | |  |
| Age | 0.310 | | 0.008 | | 0.543 | | 37.585 | | < 0.001 | | 0.294 | | 0.326 | |  |
| Age^2^ | -0.001 | | < 0.001 | | -0.040 | | -2.748 | | 0.006 | | -0.002 | | < 0.001 | |  |
| Gender | -0.132 | | 0.071 | | -0.016 | | -1.869 | | 0.062 | | -0.271 | | 0.006 | |  |
| Note. *F*(3,10234) = 1212.895, *p* < .001. *R*^2^ = .262 | | | | | | | | |  | |  | |  | |  |
|  |  | |  | |  | |  | |  | |  | |  | |  |
| UK | *b* | | *SE* | | *β* | | *t* | | *p* | | 95% LB | | 95% UB | |  |
| Age | 0.356 | | 0.006 | | 0.520 | | 54.987 | | < 0.001 | | 0.344 | | 0.369 | |  |
| Age^2^ | 0.001 | | < 0.001 | | 0.027 | | 2.822 | | 0.005 | | < 0.001 | | 0.001 | |  |
| Gender | 0.412 | | 0.051 | | 0.045 | | 8.100 | | < 0.001 | | 0.313 | | 0.512 | |  |
| Note. *F*(3,23419) = 3205.870, *p* < .001. *R*^2^ = .291 | | | | | | | | |  | |  | |  | |  |
|  |  | |  | |  | |  | |  | |  | |  | |  |
| USA | *b* | | *SE* | | *β* | | *t* | | *p* | | 95% LB | | 95% UB | |  |
| Age | 0.301 | | 0.002 | | 0.421 | | 172.613 | | < 0.001 | | 0.297 | | 0.304 | |  |
| Age^2^ | 0.003 | | < 0.001 | | 0.083 | | 34.152 | | < 0.001 | | 0.003 | | 0.003 | |  |
| Gender | 0.119 | | 0.015 | | 0.013 | | 8.142 | | < 0.001 | | 0.090 | | 0.148 | |  |
| Note. *F*(3,310023) = 32225.750, *p* < .001. *R*^2^ = .238 | | | | | | | | |  | |  | |  | |  |
| Supplementary Table 2. Results for chosen age | | | | | | | | | | | | | | | |
| Australia | | *b* | | *SE* | | *β* | | *t* | | *p* | | 95% LB | | 95% UB | |
| Age | | 0.440 | | 0.007 | | 0.634 | | 59.656 | | < 0.001 | | 0.426 | | 0.454 | |
| Age^2^ | | 0.001 | | < 0.001 | | 0.036 | | 3.384 | | 0.001 | | < 0.001 | | 0.002 | |
| Gender | | -0.442 | | 0.066 | | -0.044 | | -6.689 | | < 0.001 | | -0.572 | | -0.313 | |
| Note. *F*(3,12730) = 34440.207, *p* < .001. *R*^2^ = .448 | | | | | | | | | | | | | | | |
|  | |  | |  | |  | |  | |  | |  | |  | |
| Belgium | | *b* | | *SE* | | *β* | | *t* | | *p* | | 95% LB | | 95% UB | |
| Age | | 0.463 | | 0.018 | | 0.702 | | 25.180 | | < 0.001 | | 0.427 | | 0.499 | |
| Age^2^ | | < 0.001 | | 0.001 | | 0.007 | | 0.236 | | 0.813 | | -0.001 | | 0.002 | |
| Gender | | -0.427 | | 0.152 | | -0.044 | | -2.820 | | 0.005 | | -0.724 | | -0.130 | |
| Note. *F*(3,2053) = 724.930, *p* < .001. *R*^2^ = .514 | | | | | | | | | | | | | | | |
|  | |  | |  | |  | |  | |  | |  | |  | |
| Brazil | | *b* | | *SE* | | *β* | | *t* | | *p* | | 95% LB | | 95% UB | |
| Age | | 0.534 | | 0.016 | | 0.568 | | 33.593 | | < 0.001 | | 0.503 | | 0.565 | |
| Age^2^ | | -0.003 | | 0.001 | | -0.074 | | -4.394 | | < 0.001 | | -0.005 | | -0.002 | |
| Gender | | -0.097 | | 0.119 | | -0.009 | | -0.813 | | 0.416 | | -0.329 | | 0.136 | |
| Note. *F*(3,5393) = 657.056, *p* < .001. *R*^2^ = .268 | | | | | | | | | | | | | | | |
|  | |  | |  | |  | |  | |  | |  | |  | |
| Canada | | *b* | | *SE* | | *β* | | *t* | | *p* | | 95% LB | | 95% UB | |
| Age | | 0.474 | | 0.007 | | 0.648 | | 63.631 | | < 0.001 | | 0.459 | | 0.488 | |
| Age^2^ | | -0.001 | | < 0.001 | | -0.017 | | -1.696 | | 0.090 | | -0.001 | | < 0.001 | |
| Gender | | -0.215 | | 0.065 | | -0.021 | | -3.330 | | 0.001 | | -0.341 | | -0.088 | |
| Note. *F*(3,15332) = 3459.39, *p* < .001. *R*^2^ = .404 | | | | | | | | | | | | | | | |
|  | |  | |  | |  | |  | |  | |  | |  | |
| China | | *b* | | *SE* | | *β* | | *t* | | *p* | | 95% LB | | 95% UB | |
| Age | | 0.557 | | 0.019 | | 0.359 | | 29.969 | | < 0.001 | | 0.520 | | 0.593 | |
| Age^2^ | | -0.007 | | 0.001 | | -0.057 | | -4.769 | | < 0.001 | | -0.010 | | -0.004 | |
| Gender | | 0.482 | | 0.116 | | 0.046 | | 4.170 | | < 0.001 | | 0.255 | | 0.709 | |
| Note. *F*(3,7219) = 314.658, *p* < .001. *R*^2^ = .115 | | | | | | | | | | | | | | | |
|  | |  | |  | |  | |  | |  | |  | |  | |
| France | | *b* | | *SE* | | *β* | | *t* | | *p* | | 95% LB | | 95% UB | |
| Age | | 0.387 | | 0.008 | | 0.568 | | 47.094 | | < 0.001 | | 0.371 | | 0.404 | |
| Age^2^ | | 0.002 | | < 0.001 | | 0.065 | | 5.355 | | < 0.001 | | 0.001 | | 0.003 | |
| Gender | | -0.250 | | 0.067 | | -0.027 | | -3.748 | | < 0.001 | | -0.381 | | -0.119 | |
| Note. *F*(3,11613) = 2447.740, *p* < .001. *R*^2^ = .387 | | | | | | | | | | | | | | | |
|  | |  | |  | |  | |  | |  | |  | |  | |
| Germany | | *b* | | *SE* | | *β* | | *t* | | *p* | | 95% LB | | 95% UB | |
| Age | | 0.444 | | 0.008 | | 0.636 | | 54.271 | | < 0.001 | | 0.428 | | 0.460 | |
| Age^2^ | | -0.003 | | < 0.001 | | -0.090 | | -7.707 | | < 0.001 | | -0.004 | | -0.002 | |
| Gender | | -0.287 | | 0.063 | | -0.033 | | -4.547 | | < 0.001 | | -0.410 | | -0.163 | |
| Note. *F*(3,12749) = 2080.779, *p* < .001. *R*^2^ = .329 | | | | | | | | | | | | | | | |
|  | |  | |  | |  | |  | |  | |  | |  | |
| Korea | | *b* | | *SE* | | *β* | | *t* | | *p* | | 95% LB | | 95% UB | |
| Age | | 0.533 | | 0.019 | | 0.424 | | 27.904 | | < 0.001 | | 0.495 | | 0.570 | |
| Age^2^ | | -0.005 | | 0.001 | | -0.054 | | -3.676 | | < 0.001 | | -0.007 | | -0.002 | |
| Gender | | 0.379 | | 0.127 | | 0.036 | | 2.976 | | 0.003 | | 0.129 | | 0.629 | |
| Note. *F*(3,6159) = 358.297, *p* < .001. *R*^2^ = .149 | | | | | | | | | | | | | | | |
|  | |  | |  | |  | |  | |  | |  | |  | |
| Netherlands | | *b* | | *SE* | | *β* | | *t* | | *p* | | 95% LB | | 95% UB | |
| Age | | 0.425 | | 0.013 | | 0.646 | | 32.298 | | < 0.001 | | 0.399 | | 0.451 | |
| Age^2^ | | -0.001 | | 0.001 | | -0.022 | | -1.120 | | 0.263 | | -0.002 | | < 0.001 | |
| Gender | | -0.456 | | 0.109 | | -0.047 | | -4.198 | | < 0.001 | | -0.668 | | -0.243 | |
| Note. *F*(3,4949) = 1122.157, *p* < .001. *R*^2^ = .404 | | | | | | | | | | | | | | | |
|  | |  | |  | |  | |  | |  | |  | |  | |
| Spain | | *b* | | *SE* | | *β* | | *t* | | *p* | | 95% LB | | 95% UB | |
| Age | | 0.457 | | 0.011 | | 0.647 | | 40.575 | | < 0.001 | | 0.435 | | 0.479 | |
| Age^2^ | | < 0.001 | | < 0.001 | | < 0.001 | | 0.026 | | 0.979 | | -0.001 | | 0.001 | |
| Gender | | -0.236 | | 0.085 | | -0.024 | | -2.788 | | 0.005 | | -0.403 | | -0.070 | |
| Note. *F*(3,8166) = 2000.117, *p* < .001. *R*^2^ = .424 | | | | | | | | | | | | | | | |
|  | |  | |  | |  | |  | |  | |  | |  | |
| Sweden | | *b* | | *SE* | | *β* | | *t* | | *p* | | 95% LB | | 95% UB | |
| Age | | 0.369 | | 0.009 | | 0.567 | | 40.982 | | < 0.001 | | 0.351 | | 0.387 | |
| Age^2^ | | < 0.001 | | < 0.001 | | -0.005 | | -0.345 | | 0.730 | | -0.001 | | 0.001 | |
| Gender | | -0.606 | | 0.077 | | -0.064 | | -7.828 | | < 0.001 | | -0.757 | | -0.454 | |
| Note. *F*(3,10144) = 1625.330, *p* < .001. *R*^2^ = .325 | | | | | | | | | | | | | | | |
|  | |  | |  | |  | |  | |  | |  | |  | |
| UK | | *b* | | *SE* | | *β* | | *t* | | *p* | | 95% LB | | 95% UB | |
| Age | | 0.474 | | 0.006 | | 0.631 | | 75.712 | | < 0.001 | | 0.462 | | 0.486 | |
| Age^2^ | | 0.001 | | < 0.001 | | 0.043 | | 5.161 | | < 0.001 | | 0.001 | | 0.002 | |
| Gender | | -0.497 | | 0.049 | | -0.049 | | -10.094 | | < 0.001 | | -0.594 | | -0.401 | |
| Note. *F*(3,23228) = 6400.850, *p* < .001. *R*^2^ = .452 | | | | | | | | | | | | | | | |
|  | |  | |  | |  | |  | |  | |  | |  | |
| USA | | *b* | | *SE* | | *β* | | *t* | | *p* | | 95% LB | | 95% UB | |
| Age | | 0.515 | | 0.002 | | 0.681 | | 319.597 | | < 0.001 | | 0.511 | | 0.518 | |
| Age^2^ | | -0.002 | | < 0.001 | | -0.045 | | -21.349 | | < 0.001 | | -0.002 | | -0.001 | |
| Gender | | -0.257 | | 0.014 | | -0.026 | | -18.992 | | < 0.001 | | -0.283 | | -0.230 | |
| Note. *F*(3,309197) = 74421.466, *p* < .001. *R*^2^ = .419 | | | | | | | | | | | | | | | |

| Supplementary Table 3. Results for perceived age | | | | | | | |
| --- | --- | --- | --- | --- | --- | --- | --- |
| Australia | *b* | *SE* | *β* | *t* | *p* | 95% LB | 95% UB |
| Age | 0.219 | 0.005 | 0.561 | 45.848 | < 0.001 | 0.209 | 0.228 |
| Age^2^ | -0.001 | < 0.001 | -0.061 | -5.020 | < 0.001 | -0.001 | -0.001 |
| Gender | 0.526 | 0.043 | 0.094 | 12.299 | < 0.001 | 0.442 | 0.610 |
| Note. *F*(3,12785) = 1512.289, *p* < .001. *R*^2^ = .262 | | | | | | | |
|  |  |  |  |  |  |  |  |
| Belgium | *b* | *SE* | *β* | *t* | *p* | 95% LB | 95% UB |
| Age | 0.183 | 0.009 | 0.648 | 19.713 | < 0.001 | 0.165 | 0.201 |
| Age^2^ | -0.001 | < 0.001 | -0.094 | -2.882 | 0.004 | -0.002 | 0.000 |
| Gender | 0.226 | 0.077 | 0.054 | 2.941 | 0.003 | 0.075 | 0.378 |
| Note. *F*(3,2067) = 322.068, *p* < .001. *R*^2^ = .319 | | | | | | | |
|  |  |  |  |  |  |  |  |
| Brazil | *b* | *SE* | *β* | *t* | *p* | 95% LB | 95% UB |
| Age | 0.285 | 0.007 | 0.656 | 40.328 | < 0.001 | 0.271 | 0.298 |
| Age^2^ | -0.004 | < 0.001 | -0.178 | -10.933 | < 0.001 | -0.004 | -0.003 |
| Gender | 0.909 | 0.053 | 0.194 | 17.263 | < 0.001 | 0.806 | 1.012 |
| Note. *F*(3,5415) = 852.637, *p* < .001. *R*^2^ = .321 | | | | | | | |
|  |  |  |  |  |  |  |  |
| Canada | *b* | *SE* | *β* | *t* | *p* | 95% LB | 95% UB |
| Age | 0.271 | 0.004 | 0.672 | 60.879 | < 0.001 | 0.262 | 0.279 |
| Age^2^ | -0.003 | < 0.001 | -0.178 | -16.130 | < 0.001 | -0.004 | -0.003 |
| Gender | 0.476 | 0.039 | 0.083 | 12.352 | < 0.001 | 0.401 | 0.552 |
| Note. *F*(3,15408) = 2181.882, *p* < .001. *R*^2^ = .298 | | | | | | | |
|  |  |  |  |  |  |  |  |
| China | *b* | *SE* | *β* | *t* | *p* | 95% LB | 95% UB |
| Age | 0.287 | 0.008 | 0.408 | 35.087 | < 0.001 | 0.271 | 0.303 |
| Age^2^ | -0.003 | 0.001 | -0.048 | -4.166 | < 0.001 | -0.004 | -0.001 |
| Gender | 0.674 | 0.051 | 0.143 | 13.242 | < 0.001 | 0.575 | 0.774 |
| Note. *F*(3,7252) = 468.428, *p* < .001. *R*^2^ = .162 | | | | | | | |
|  |  |  |  |  |  |  |  |
| France | *b* | *SE* | *β* | *t* | *p* | 95% LB | 95% UB |
| Age | 0.211 | 0.004 | 0.654 | 50.797 | < 0.001 | 0.203 | 0.219 |
| Age^2^ | -0.002 | < 0.001 | -0.145 | -11.297 | < 0.001 | -0.003 | -0.002 |
| Gender | 0.321 | 0.034 | 0.074 | 9.502 | < 0.001 | 0.255 | 0.387 |
| Note. *F*(3,11670) = 1662.615, *p* < .001. *R*^2^ = .299 | | | | | | | |
|  |  |  |  |  |  |  |  |
| Germany | *b* | *SE* | *β* | *t* | *p* | 95% LB | 95% UB |
| Age | 0.243 | 0.004 | 0.691 | 57.234 | < 0.001 | 0.235 | 0.252 |
| Age^2^ | -0.004 | < 0.001 | -0.221 | -18.371 | < 0.001 | -0.004 | -0.003 |
| Gender | 0.475 | 0.033 | 0.109 | 14.491 | < 0.001 | 0.411 | 0.540 |
| Note. *F*(3,12811) = 1709.881, *p* < .001. *R*^2^ = .286 | | | | | | | |
|  |  |  |  |  |  |  |  |
| Korea | *b* | *SE* | *β* | *t* | *p* | 95% LB | 95% UB |
| Age | 0.261 | 0.012 | 0.350 | 22.234 | < 0.001 | 0.238 | 0.284 |
| Age^2^ | -0.003 | 0.001 | -0.050 | -3.261 | 0.001 | -0.004 | -0.001 |
| Gender | 0.642 | 0.078 | 0.103 | 8.195 | < 0.001 | 0.488 | 0.795 |
| Note. *F*(3,6140) = 218.807, *p* < .001. *R*^2^ = .096 | | | | | | | |
|  |  |  |  |  |  |  |  |
| Netherlands | *b* | *SE* | *β* | *t* | *p* | 95% LB | 95% UB |
| Age | 0.187 | 0.006 | 0.663 | 30.814 | < 0.001 | 0.176 | 0.199 |
| Age^2^ | -0.001 | < 0.001 | -0.123 | -5.726 | < 0.001 | -0.002 | -0.001 |
| Gender | 0.310 | 0.050 | 0.074 | 6.184 | < 0.001 | 0.212 | 0.408 |
| Note. *F*(3,4985) = 748.486, *p* < .001. *R*^2^ = .310 | | | | | | | |
|  |  |  |  |  |  |  |  |
| Spain | *b* | *SE* | *β* | *t* | *p* | 95% LB | 95% UB |
| Age | 0.378 | 0.011 | 0.574 | 32.896 | < 0.001 | 0.356 | 0.401 |
| Age^2^ | < 0.001 | < 0.001 | -0.013 | -0.729 | 0.466 | -0.001 | 0.001 |
| Gender | -0.315 | 0.086 | -0.034 | -3.651 | < 0.001 | -0.485 | -0.146 |
| Note. *F*(3,7787) = 1237.245, *p* < .001. *R*^2^ = .323 | | | | | | | |
|  |  |  |  |  |  |  |  |
| Sweden | *b* | *SE* | *β* | *t* | *p* | 95% LB | 95% UB |
| Age | 0.244 | 0.005 | 0.740 | 53.458 | < 0.001 | 0.235 | 0.253 |
| Age^2^ | -0.003 | < 0.001 | -0.230 | -16.607 | < 0.001 | -0.003 | -0.003 |
| Gender | 0.235 | 0.039 | 0.049 | 5.984 | < 0.001 | 0.158 | 0.312 |
| Note. *F*(3,10166) = 1633.271, *p* < .001. *R*^2^ = .325 | | | | | | | |
|  |  |  |  |  |  |  |  |
| UK | *b* | *SE* | *β* | *t* | *p* | 95% LB | 95% UB |
| Age | 0.232 | 0.004 | 0.608 | 62.955 | < 0.001 | 0.225 | 0.240 |
| Age^2^ | -0.002 | < 0.001 | -0.132 | -13.685 | < 0.001 | -0.002 | -0.002 |
| Gender | 0.688 | 0.029 | 0.134 | 23.717 | < 0.001 | 0.631 | 0.745 |
| Note. *F*(3,23300)= 2755.206, *p* < .001. *R*^2^ = .262 | | | | | | | |
|  |  |  |  |  |  |  |  |
| USA | *b* | *SE* | *β* | *t* | *p* | 95% LB | 95% UB |
| Age | 0.310 | 0.001 | 0.677 | 291.921 | < 0.001 | 0.308 | 0.312 |
| Age^2^ | -0.004 | < 0.001 | -0.183 | -78.997 | < 0.001 | -0.004 | -0.004 |
| Gender | 0.613 | 0.009 | 0.103 | 68.744 | < 0.001 | 0.596 | 0.631 |
| Note. *F*(3,309375) = 46411.690, *p* < .001. *R*^2^ = .310 | | | | | | | |

| Supplementary Table 4. Results for age they hope to live until | | | | | |  |  |
| --- | --- | --- | --- | --- | --- | --- | --- |
| Australia | *b* | *SE* | *β* | *t* | *p* | 95% LB | 95% UB |
| Age | 0.024 | 0.017 | 0.020 | 1.393 | 0.164 | -0.010 | 0.057 |
| Age^2^ | < 0.001 | 0.001 | 0.001 | 0.075 | 0.941 | -0.001 | 0.002 |
| Gender | -0.131 | 0.151 | -0.008 | -0.866 | 0.387 | -0.427 | 0.165 |
| Note. *F*(3,12752) = 2.252, *p* =.080. *R*^2^ = .001 | | | | |  |  |  |
|  |  |  |  |  |  |  |  |
| Belgium | *b* | *SE* | *β* | *t* | *p* | 95% LB | 95% UB |
| Age | -0.011 | 0.041 | -0.011 | -0.272 | 0.786 | -0.091 | 0.069 |
| Age^2^ | 0.001 | 0.002 | 0.031 | 0.788 | 0.431 | -0.002 | 0.004 |
| Gender | 0.448 | 0.337 | 0.030 | 1.328 | 0.184 | -0.214 | 1.11 |
| Note. *F*(3,2052) = 0.871, *p* = .456. *R*^2^ = .001 | | | | | | | |
|  |  |  |  |  |  |  |  |
| Brazil | *b* | *SE* | *β* | *t* | *p* | 95% LB | 95% UB |
| Age | 0.051 | 0.030 | 0.034 | 1.695 | 0.090 | -0.008 | 0.110 |
| Age^2^ | -0.001 | 0.001 | -0.017 | -0.843 | 0.399 | -0.004 | 0.002 |
| Gender | -0.602 | 0.225 | -0.036 | -2.677 | 0.007 | -1.043 | -0.161 |
| Note. *F*(3,5410) = 3.588, *p* < .001. *R*^2^= .002 | | | | | | | |
|  |  |  |  |  |  |  |  |
| Canada | *b* | *SE* | *β* | *t* | *p* | 95% LB | 95% UB |
| Age | 0.005 | 0.016 | 0.004 | 0.294 | 0.769 | -0.027 | 0.036 |
| Age^2^ | < 0.001 | 0.001 | 0.003 | 0.258 | 0.797 | -0.001 | 0.002 |
| Gender | -0.226 | 0.140 | -0.013 | -1.610 | 0.107 | -0.501 | 0.049 |
| Note. *F*(3,15367) = 1.132, *p* = .335 *R*^2^ = 0 | | | |  |  |  |  |
|  |  |  |  |  |  |  |  |
| China | *b* | *SE* | *β* | *t* | *p* | 95% LB | 95% UB |
| Age | 0.031 | 0.035 | 0.011 | 0.880 | 0.379 | -0.038 | 0.101 |
| Age^2^ | 0.003 | 0.003 | 0.013 | 1.053 | 0.292 | -0.003 | 0.009 |
| Gender | -2.809 | 0.220 | -0.149 | -12.745 | < 0.001 | -3.242 | -2.377 |
| Note. *F*(3,7227) =56.598, *p* < .001. *R*^2^= .023 | | | | |  |  |  |
|  |  |  |  |  |  |  |  |
| France | *b* | *SE* | *β* | *t* | *p* | 95% LB | 95% UB |
| Age | -0.018 | 0.018 | -0.015 | -0.998 | 0.318 | -0.054 | 0.018 |
| Age^2^ | < 0.001 | 0.001 | 0.001 | 0.059 | 0.953 | -0.002 | 0.002 |
| Gender | -0.099 | 0.148 | -0.006 | -0.665 | 0.506 | -0.390 | 0.192 |
| Note. *F*(3,11599) = 0.958 *p* = .412. *R*^2^= 0 | | | |  |  |  |  |
|  |  |  |  |  |  |  |  |
| Germany | *b* | *SE* | *β* | *t* | *p* | 95% LB | 95% UB |
| Age | 0.043 | 0.019 | 0.032 | 2.231 | 0.026 | 0.005 | 0.081 |
| Age^2^ | -0.002 | 0.001 | -0.029 | -2.020 | 0.043 | -0.004 | 0.000 |
| Gender | -1.386 | 0.149 | -0.083 | -9.326 | < 0.001 | -1.677 | -1.094 |
| Note. *F*(3,12735) = 32.398, *p* < .001. *R*^2^= 0.008 | | | | |  |  |  |
|  |  |  |  |  |  |  |  |
| Korea | *b* | *SE* | *β* | *t* | *p* | 95% LB | 95% UB |
| Age | -0.037 | 0.040 | -0.015 | -0.906 | 0.365 | -0.116 | 0.043 |
| Age^2^ | 0.004 | 0.003 | 0.024 | 1.498 | 0.134 | -0.001 | 0.010 |
| Gender | -2.720 | 0.270 | -0.132 | -10.072 | < 0.001 | -3.249 | -2.19 |
| Note. *F*(3,6153) = 36.796, *p* < .001. *R*^2^= .018 | | | | |  |  |  |
|  |  |  |  |  |  |  |  |
| Netherlands | *b* | *SE* | *β* | *t* | *p* | 95% LB | 95% UB |
| Age | -0.038 | 0.025 | -0.04 | -1.527 | 0.127 | -0.088 | 0.011 |
| Age^2^ | 0.001 | 0.001 | 0.031 | 1.196 | 0.232 | -0.001 | 0.003 |
| Gender | -0.613 | 0.207 | -0.043 | -2.964 | 0.003 | -1.018 | -0.208 |
| Note. *F*(3,4962) = 3.616, *p* < .001. *R*^2^= .002 | | | | | | | |
|  |  |  |  |  |  |  |  |
| Spain | *b* | *SE* | *β* | *t* | *p* | 95% LB | 95% UB |
| Age | -0.115 | 0.022 | -0.107 | -5.132 | < 0.001 | -0.159 | -0.071 |
| Age^2^ | 0.004 | 0.001 | 0.090 | 4.309 | < 0.001 | 0.002 | 0.005 |
| Gender | 0.250 | 0.169 | 0.017 | 1.480 | 0.139 | -0.081 | 0.581 |
| Note. *F*(3,8174) = 10.402, *p* < .001. *R*^2^= .004 | | | | |  |  |  |
|  |  |  |  |  |  |  |  |
| Sweden | *b* | *SE* | *β* | *t* | *p* | 95% LB | 95% UB |
| Age | 0.019 | 0.018 | 0.018 | 1.067 | 0.286 | -0.016 | 0.054 |
| Age^2^ | -0.003 | 0.001 | -0.060 | -3.528 | < 0.001 | -0.004 | -0.001 |
| Gender | -0.712 | 0.153 | -0.046 | -4.636 | < 0.001 | -1.012 | -0.411 |
| Note. *F*(3,10116) = 14.255, *p* < .001. *R*^2^= .004 | | | | |  |  |  |
|  |  |  |  |  |  |  |  |
| UK | *b* | *SE* | *β* | *t* | *p* | 95% LB | 95% UB |
| Age | -0.001 | 0.012 | -0.001 | -0.088 | 0.930 | -0.025 | 0.023 |
| Age^2^ | < 0.001 | 0.001 | 0.007 | 0.640 | 0.522 | -0.001 | 0.001 |
| Gender | -0.329 | 0.096 | -0.023 | -3.419 | 0.001 | -0.517 | -0.140 |
| Note. *F*(3,23213) = 4.348, *p* < .001. *R*^2^= .001 | | | | |  |  |  |
|  |  |  |  |  |  |  |  |
| USA | *b* | *SE* | *β* | *t* | *p* | 95% LB | 95% UB |
| Age | -0.073 | 0.004 | -0.057 | -20.326 | < 0.001 | -0.08 | -0.066 |
| Age^2^ | 0.003 | < 0.001 | 0.043 | 15.353 | < 0.001 | 0.002 | 0.003 |
| Gender | 0.177 | 0.030 | 0.011 | 5.890 | < 0.001 | 0.118 | 0.236 |
| Note. *F*(3,309086) = 151.063, *p* < .001. *R*^2^ = .001 | | | | |  |  |  |

| Supplementary Table 5. Results for childhood to young adult transition | | | | | | | |
| --- | --- | --- | --- | --- | --- | --- | --- |
| Australia | *b* | *SE* | *β* | *t* | *p* | 95% LB | 95% UB |
| Age | 0.025 | 0.003 | 0.113 | 7.971 | < 0.001 | 0.019 | 0.032 |
| Age^2^ | < 0.001 | < 0.001 | -0.019 | -1.348 | 0.178 | < 0.001 | < 0.001 |
| Gender | 0.013 | 0.029 | 0.004 | 0.454 | 0.650 | -0.043 | 0.069 |
| Note. *F*(3,12810) = 41.796, *p* < .001. *R*^2^= .01 | | | | |  |  |  |
|  |  |  |  |  |  |  |  |
| Belgium | *b* | *SE* | *β* | *t* | *p* | 95% LB | 95% UB |
| Age | 0.019 | 0.007 | 0.113 | 2.873 | 0.004 | 0.006 | 0.033 |
| Age^2^ | < 0.001 | < 0.001 | 0.005 | 0.138 | 0.890 | < 0.001 | 0.001 |
| Gender | -0.027 | 0.056 | -0.011 | -0.476 | 0.634 | -0.136 | 0.083 |
| Note. *F*(3,2075) = 10.111, *p* < .001. *R*^2^= .014 | | | | |  |  |  |
|  |  |  |  |  |  |  |  |
| Brazil | *b* | *SE* | *β* | *t* | *p* | 95% LB | 95% UB |
| Age | 0.005 | 0.007 | 0.014 | 0.721 | 0.471 | -0.009 | 0.019 |
| Age^2^ | < 0.001 | < 0.001 | < 0.001 | 0.010 | 0.992 | -0.001 | 0.001 |
| Gender | 0.067 | 0.052 | 0.018 | 1.292 | 0.196 | -0.035 | 0.169 |
| Note. *F*(3,5434) = 0.888, *p* = .446. *R*^2^= 0 | | | |  |  |  |  |
|  |  |  |  |  |  |  |  |
| Canada | *b* | *SE* | *β* | *t* | *p* | 95% LB | 95% UB |
| Age | 0.028 | 0.003 | 0.117 | 8.907 | < 0.001 | 0.022 | 0.034 |
| Age^2^ | < 0.001 | < 0.001 | -0.018 | -1.377 | 0.169 | < 0.001 | < 0.001 |
| Gender | 0.066 | 0.027 | 0.02 | 2.438 | 0.015 | 0.013 | 0.119 |
| Note. *F*(3,15453) = 56.522, *p* < .001. *R*^2^= .011 | | | | |  |  |  |
|  |  |  |  |  |  |  |  |
| China | *b* | *SE* | *β* | *t* | *p* | 95% LB | 95% UB |
| Age | 0.009 | 0.004 | 0.026 | 2.033 | 0.042 | 0.000 | 0.018 |
| Age^2^ | -0.001 | < 0.001 | -0.021 | -1.670 | 0.095 | -0.001 | < 0.001 |
| Gender | 0.056 | 0.028 | 0.024 | 2.004 | 0.045 | 0.001 | 0.111 |
| Note. *F*(3,7290) = 2.743, *p* = .042. *R*^2^=.001 | | | | | | | |
|  |  |  |  |  |  |  |  |
| France | *b* | *SE* | *β* | *t* | *p* | 95% LB | 95% UB |
| Age | 0.041 | 0.005 | 0.115 | 7.491 | < 0.001 | 0.030 | 0.052 |
| Age^2^ | < 0.001 | < 0.001 | 0.001 | 0.036 | 0.971 | < 0.001 | 0.001 |
| Gender | 0.099 | 0.044 | 0.020 | 2.224 | 0.026 | 0.012 | 0.185 |
| Note. *F*(3,11690) = 53.164, *p* < .001. *R*^2^= .013 | | | | |  |  |  |
|  |  |  |  |  |  |  |  |
| Germany | *b* | *SE* | *β* | *t* | *p* | 95% LB | 95% UB |
| Age | -0.012 | 0.002 | -0.087 | -6.152 | < 0.001 | -0.016 | -0.008 |
| Age^2^ | < 0.001 | < 0.001 | 0.058 | 4.077 | < 0.001 | < 0.001 | 0.001 |
| Gender | < 0.001 | 0.016 | 0.000 | 0.001 | 0.999 | -0.03 | 0.031 |
| Note. *F*(3,12943) = 13.246, *p* < .001. *R*^2^= .003 | | | | |  |  |  |
|  |  |  |  |  |  |  |  |
| Korea | *b* | *SE* | *β* | *t* | *p* | 95% LB | 95% UB |
| Age | 0.039 | 0.006 | 0.102 | 6.235 | < 0.001 | 0.027 | 0.052 |
| Age^2^ | < 0.001 | < 0.001 | -0.010 | -0.648 | 0.517 | -0.001 | 0.001 |
| Gender | -0.002 | 0.042 | -0.001 | -0.056 | 0.955 | -0.085 | 0.080 |
| Note. *F*(3,6166) = 19.324, *p* < .001. *R*^2^= .009 | | | | |  |  |  |
|  |  |  |  |  |  |  |  |
| Netherlands | *b* | *SE* | *β* | *t* | *p* | 95% LB | 95% UB |
| Age | 0.015 | 0.005 | 0.073 | 2.832 | 0.005 | 0.005 | 0.025 |
| Age^2^ | < 0.001 | < 0.001 | 0.006 | 0.225 | 0.822 | < 0.001 | < 0.001 |
| Gender | 0.033 | 0.044 | 0.011 | 0.744 | 0.457 | -0.053 | 0.118 |
| Note. *F*(3,4997) = 9.944, *p* < .001. *R*^2^= .006 | | | | | | | |
|  |  |  |  |  |  |  |  |
| Spain | *b* | *SE* | *β* | *t* | *p* | 95% LB | 95% UB |
| Age | 0.013 | 0.004 | 0.064 | 3.064 | 0.002 | 0.005 | 0.021 |
| Age^2^ | < 0.001 | < 0.001 | -0.056 | -2.700 | 0.007 | -0.001 | < 0.001 |
| Gender | 0.196 | 0.031 | 0.069 | 6.228 | < 0.001 | 0.134 | 0.257 |
| Note. *F*(3,8245) = 14.649, *p* < .001. *R*^2^= .005 | | | | |  |  |  |
|  |  |  |  |  |  |  |  |
| Sweden | *b* | *SE* | *β* | *t* | *p* | 95% LB | 95% UB |
| Age | -0.015 | 0.003 | -0.087 | -5.179 | < 0.001 | -0.020 | -0.009 |
| Age^2^ | 0.001 | < 0.001 | 0.092 | 5.478 | < 0.001 | < 0.001 | 0.001 |
| Gender | -0.004 | 0.024 | -0.002 | -0.155 | 0.877 | -0.052 | 0.044 |
| Note. *F*(3,10235) = 10.543, *p* < .001. *R*^2^= .003 | | | | |  |  |  |
|  |  |  |  |  |  |  |  |
| UK | *b* | *SE* | *β* | *t* | *p* | 95% LB | 95% UB |
| Age | 0.012 | 0.002 | 0.052 | 4.679 | < 0.001 | 0.007 | 0.017 |
| Age^2^ | < 0.001 | < 0.001 | 0.008 | 0.709 | 0.478 | < 0.001 | < 0.001 |
| Gender | 0.071 | 0.020 | 0.024 | 3.615 | < 0.001 | 0.032 | 0.109 |
| Note. *F*(3,23397) = 29.82, *p* < .001. *R*^2^= .004 | | | | |  |  |  |
|  |  |  |  |  |  |  |  |
| USA | *b* | *SE* | *β* | *t* | *p* | 95% LB | 95% UB |
| Age | 0.044 | 0.001 | 0.165 | 59.618 | < 0.001 | 0.042 | 0.045 |
| Age^2^ | -0.001 | < 0.001 | -0.055 | -19.825 | < 0.001 | -0.001 | -0.001 |
| Gender | 0.106 | 0.006 | 0.031 | 17.251 | < 0.001 | 0.094 | 0.118 |
| Note. *F*(3,308142) = 1800.063, *p* < .001. *R*^2^= .017 | | | | |  |  |  |

| Supplementary Table 6. Results for young adult to adult transition | | | | | |  |  |
| --- | --- | --- | --- | --- | --- | --- | --- |
| Australia | *b* | *SE* | *β* | *t* | *p* | 95% LB | 95% UB |
| Age | 0.028 | 0.004 | 0.098 | 6.893 | < 0.001 | 0.020 | 0.037 |
| Age^2^ | < 0.001 | < 0.001 | 0.029 | 2.043 | 0.041 | < 0.001 | 0.001 |
| Gender | 0.157 | 0.037 | 0.037 | 4.236 | < 0.001 | 0.084 | 0.229 |
| Note. *F*(3,12796) = 65.216, *p* < .001. *R*^2^= .015 | | | | |  |  |  |
|  |  |  |  |  |  |  |  |
| Belgium | *b* | *SE* | *β* | *t* | *p* | 95% LB | 95% UB |
| Age | 0.039 | 0.011 | 0.144 | 3.665 | < 0.001 | 0.018 | 0.060 |
| Age^2^ | < 0.001 | < 0.001 | 0.005 | 0.124 | 0.901 | -0.001 | 0.001 |
| Gender | 0.086 | 0.088 | 0.022 | 0.980 | 0.327 | -0.086 | 0.258 |
| Note. *F*(3,2072) = 14.965, *p* < .001. *R*^2^= 0.021 | | | | |  |  |  |
|  |  |  |  |  |  |  |  |
| Brazil | *b* | *SE* | *β* | *t* | *p* | 95% LB | 95% UB |
| Age | 0.014 | 0.009 | 0.030 | 1.532 | 0.126 | -0.004 | 0.032 |
| Age^2^ | < 0.001 | < 0.001 | < 0.001 | < 0.001 | 1.000 | -0.001 | 0.001 |
| Gender | 0.201 | 0.068 | 0.040 | 2.967 | 0.003 | 0.068 | 0.333 |
| Note. *F*(3,5428) = 4.388, *p* <.004. *R*^2^= 0.002 | | | |  |  |  |  |
|  |  |  |  |  |  |  |  |
| Canada | *b* | *SE* | *β* | *t* | *p* | 95% LB | 95% UB |
| Age | 0.051 | 0.004 | 0.163 | 12.463 | < 0.001 | 0.043 | 0.059 |
| Age^2^ | -0.001 | < 0.001 | -0.064 | -4.914 | < 0.001 | -0.001 | -0.001 |
| Gender | 0.146 | 0.036 | 0.033 | 4.102 | < 0.001 | 0.076 | 0.215 |
| Note. *F*(3,15424) = 78.041, *p* < .001. *R*^2^= 0.15 | | | | |  |  |  |
|  |  |  |  |  |  |  |  |
| China | *b* | *SE* | *β* | *t* | *p* | 95% LB | 95% UB |
| Age | 0.026 | 0.006 | 0.058 | 4.559 | < 0.001 | 0.015 | 0.037 |
| Age^2^ | -0.002 | < 0.001 | -0.050 | -3.996 | < 0.001 | -0.003 | -0.001 |
| Gender | 0.075 | 0.036 | 0.025 | 2.098 | 0.036 | 0.005 | 0.144 |
| Note. *F*(3,7277) = 9.789, *p* < .042. *R*^2^= 0.004 | | | | | | | |
|  |  |  |  |  |  |  |  |
| France | *b* | *SE* | *β* | *t* | *p* | 95% LB | 95% UB |
| Age | 0.043 | 0.006 | 0.102 | 6.653 | < 0.001 | 0.030 | 0.056 |
| Age^2^ | < 0.001 | < 0.001 | -0.004 | -0.271 | 0.787 | -0.001 | 0.001 |
| Gender | 0.340 | 0.053 | 0.059 | 6.456 | < 0.001 | 0.237 | 0.444 |
| Note. *F*(3,11669) = 50.367, *p* < .001. *R*^2^= 0.013 | | | | |  |  |  |
|  |  |  |  |  |  |  |  |
| Germany | *b* | *SE* | *β* | *t* | *p* | 95% LB | 95% UB |
| Age | 0.016 | 0.003 | 0.078 | 5.460 | < 0.001 | 0.010 | 0.021 |
| Age^2^ | -0.001 | < 0.001 | -0.064 | -4.491 | < 0.001 | -0.001 | 0.000 |
| Gender | 0.049 | 0.022 | 0.019 | 2.201 | 0.028 | 0.005 | 0.093 |
| Note. *F*(3,12905) = 10.918, *p* < .001. *R*^2^= 003 | | | | |  |  |  |
|  |  |  |  |  |  |  |  |
| Korea | *b* | *SE* | *β* | *t* | *p* | 95% LB | 95% UB |
| Age | 0.102 | 0.009 | 0.176 | 10.806 | < 0.001 | 0.083 | 0.120 |
| Age^2^ | -0.002 | 0.001 | -0.052 | -3.301 | 0.001 | -0.003 | -0.001 |
| Gender | -0.178 | 0.063 | -0.037 | -2.825 | 0.005 | -0.301 | -0.054 |
| Note. *F*(3,6162) = 56.178, *p* < .001. *R*^2^= 0.027 | | | | |  |  |  |
|  |  |  |  |  |  |  |  |
| Netherlands | *b* | *SE* | *β* | *t* | *p* | 95% LB | 95% UB |
| Age | 0.026 | 0.007 | 0.089 | 3.454 | 0.001 | 0.011 | 0.040 |
| Age^2^ | < 0.001 | < 0.001 | -0.013 | -0.515 | 0.606 | -0.001 | 0.000 |
| Gender | 0.075 | 0.061 | 0.018 | 1.232 | 0.218 | -0.044 | 0.195 |
| Note. *F*(3,4991) = 10.067, *p* < .001. *R*^2^=0.006 | | | | |  |  |  |
|  |  |  |  |  |  |  |  |
| Spain | *b* | *SE* | *β* | *t* | *p* | 95% LB | 95% UB |
| Age | 0.041 | 0.008 | 0.102 | 4.854 | < 0.001 | 0.024 | 0.057 |
| Age^2^ | -0.002 | 0.000 | -0.100 | -4.783 | < 0.001 | -0.002 | -0.001 |
| Gender | 0.537 | 0.063 | 0.094 | 8.527 | < 0.001 | 0.414 | 0.661 |
| Note. *F*(3,8238) = 29.649, *p* < .001. *R*^2^= 0.011 | | | | |  |  |  |
|  |  |  |  |  |  |  |  |
| Sweden | *b* | *SE* | *β* | *t* | *p* | 95% LB | 95% UB |
| Age | 0.001 | 0.004 | 0.003 | 0.171 | 0.865 | -0.008 | 0.009 |
| Age^2^ | < 0.001 | < 0.001 | 0.010 | 0.619 | 0.536 | < 0.001 | < 0.001 |
| Gender | 0.040 | 0.037 | 0.011 | 1.086 | 0.278 | -0.032 | 0.112 |
| Note. *F*(3,10220) = 0.932, *p* = .424. *R*^2^= 0 | | | |  |  |  |  |
|  |  |  |  |  |  |  |  |
| UK | *b* | *SE* | *β* | *t* | *p* | 95% LB | 95% UB |
| Age | -0.007 | 0.003 | -0.023 | -2.06 | 0.039 | -0.014 | < 0.001 |
| Age^2^ | 0.001 | < 0.001 | 0.067 | 5.972 | < 0.001 | < 0.001 | 0.001 |
| Gender | 0.185 | 0.026 | 0.046 | 6.988 | < 0.001 | 0.133 | 0.237 |
| Note. *F*(3,23355) = 35.129, *p* < .001. *R*^2^= 0.004 | | | | |  |  |  |
|  |  |  |  |  |  |  |  |
| USA | *b* | *SE* | *β* | *t* | *p* | 95% LB | 95% UB |
| Age | 0.067 | 0.001 | 0.190 | 68.612 | < 0.001 | 0.065 | 0.068 |
| Age^2^ | -0.001 | < 0.001 | -0.063 | -22.840 | < 0.001 | -0.001 | -0.001 |
| Gender | 0.163 | 0.008 | 0.036 | 20.071 | < 0.001 | 0.147 | 0.179 |
| Note. *F*(3,307670) = 2384.676, *p* < .001. *R*^2^= 0.023 | | | | |  |  |  |

| Supplementary Table 7. Results for adult to middle-age transition | | | | | |  |  |
| --- | --- | --- | --- | --- | --- | --- | --- |
| Australia | *b* | *SE* | *β* | *t* | *p* | 95% LB | 95% UB |
| Age | 0.250 | 0.007 | 0.466 | 35.452 | < 0.001 | 0.237 | 0.264 |
| Age^2^ | -0.003 | < 0.001 | -0.114 | -8.708 | < 0.001 | -0.003 | -0.002 |
| Gender | 1.028 | 0.063 | 0.133 | 16.243 | < 0.001 | 0.904 | 1.152 |
| Note. *F*(3,12798) = 764.956, *p* < .001. *R*^2^= .152 | | | | |  |  |  |
|  |  |  |  |  |  |  |  |
| Belgium | *b* | *SE* | *β* | *t* | *p* | 95% LB | 95% UB |
| Age | 0.069 | 0.012 | 0.234 | 5.983 | < 0.001 | 0.047 | 0.092 |
| Age^2^ | -0.001 | < 0.001 | -0.116 | -2.989 | 0.003 | -0.002 | < 0.001 |
| Gender | 0.304 | 0.096 | 0.07 | 3.174 | 0.002 | 0.116 | 0.493 |
| Note. *F*(3,2071) = 17.228, *p* < .001. *R*^2^= .024 | | | | | | | |
|  |  |  |  |  |  |  |  |
| Brazil | *b* | *SE* | *β* | *t* | *p* | 95% LB | 95% UB |
| Age | 0.115 | 0.014 | 0.157 | 8.136 | < 0.001 | 0.088 | 0.143 |
| Age^2^ | -0.002 | 0.001 | -0.046 | -2.377 | 0.017 | -0.003 | < 0.001 |
| Gender | 1.357 | 0.106 | 0.170 | 12.834 | < 0.001 | 1.150 | 1.565 |
| Note. *F*(3,5432) = 82.215, *p* < .001. *R*^2^= .043 | | | | | | | |
|  |  |  |  |  |  |  |  |
| Canada | *b* | *SE* | *β* | *t* | *p* | 95% LB | 95% UB |
| Age | 0.233 | 0.008 | 0.376 | 29.918 | < 0.001 | 0.218 | 0.249 |
| Age^2^ | -0.004 | < 0.001 | -0.146 | -11.591 | < 0.001 | -0.005 | -0.003 |
| Gender | 1.025 | 0.068 | 0.116 | 15.137 | < 0.001 | 0.892 | 1.157 |
| Note. *F*(3,15437) = 494.288, *p* < .001. *R*^2^= .0.88 | | | | |  |  |  |
|  |  |  |  |  |  |  |  |
| China | *b* | *SE* | *β* | *t* | *p* | 95% LB | 95% UB |
| Age | 0.197 | 0.012 | 0.206 | 16.605 | < 0.001 | 0.174 | 0.22 |
| Age^2^ | -0.002 | 0.001 | -0.021 | -1.679 | 0.093 | -0.003 | < 0.001 |
| Gender | 0.590 | 0.074 | 0.092 | 7.982 | < 0.001 | 0.445 | 0.735 |
| Note. *F*(3,7269) = 111.941, *p* < .001. *R*^2^ = .044 | | | | |  |  |  |
|  |  |  |  |  |  |  |  |
| France | *b* | *SE* | *β* | *t* | *p* | 95% LB | 95% UB |
| Age | 0.259 | 0.013 | 0.302 | 20.110 | < 0.001 | 0.234 | 0.284 |
| Age^2^ | -0.007 | 0.001 | -0.165 | -11.025 | < 0.001 | -0.008 | -0.005 |
| Gender | 1.365 | 0.104 | 0.118 | 13.072 | < 0.001 | 1.160 | 1.570 |
| Note. *F*(3,11671) = 207.568, *p* < .001. *R*^2^= .051 | | | | |  |  |  |
|  |  |  |  |  |  |  |  |
| Germany | *b* | *SE* | *β* | *t* | *p* | 95% LB | 95% UB |
| Age | 0.087 | 0.008 | 0.155 | 11.106 | < 0.001 | 0.072 | 0.103 |
| Age^2^ | -0.002 | < 0.001 | -0.091 | -6.529 | < 0.001 | -0.003 | -0.002 |
| Gender | 1.131 | 0.061 | 0.163 | 18.669 | < 0.001 | 1.013 | 1.250 |
| Note. *F*(3,12920) = 147.067, *p* < .001. *R*^2^= .033 | | | | |  |  |  |
|  |  |  |  |  |  |  |  |
| Korea | *b* | *SE* | *β* | *t* | *p* | 95% LB | 95% UB |
| Age | 0.131 | 0.013 | 0.170 | 10.399 | < 0.001 | 0.106 | 0.155 |
| Age^2^ | -0.002 | 0.001 | -0.036 | -2.261 | 0.024 | -0.004 | < 0.001 |
| Gender | 0.175 | 0.084 | 0.027 | 2.081 | 0.038 | 0.010 | 0.339 |
| Note. *F*(3,6164) = 44.518, *p* < .001. *R*^2^= .021 | | | | | | | |
|  |  |  |  |  |  |  |  |
| Netherlands | *b* | *SE* | *β* | *t* | *p* | 95% LB | 95% UB |
| Age | 0.067 | 0.008 | 0.208 | 8.143 | < 0.001 | 0.051 | 0.083 |
| Age^2^ | -0.001 | < 0.001 | -0.058 | -2.256 | 0.024 | -0.001 | < 0.001 |
| Gender | 0.179 | 0.068 | 0.037 | 2.633 | 0.008 | 0.046 | 0.313 |
| Note. *F*(3,4991) = 44.647, *p* < .001. *R*^2^= .026 | | | | | | | |
|  |  |  |  |  |  |  |  |
| Spain | *b* | *SE* | *β* | *t* | *p* | 95% LB | 95% UB |
| Age | 0.313 | 0.017 | 0.372 | 18.526 | < 0.001 | 0.280 | 0.347 |
| Age^2^ | -0.004 | 0.001 | -0.131 | -6.529 | < 0.001 | -0.005 | -0.003 |
| Gender | 1.544 | 0.128 | 0.129 | 12.098 | < 0.001 | 1.294 | 1.794 |
| Note. *F*(3,8235) = 241.397, *p* < .001. *R*^2^= .081 | | | | | | | |
|  |  |  |  |  |  |  |  |
| Sweden | *b* | *SE* | *β* | *t* | *p* | 95% LB | 95% UB |
| Age | 0.190 | 0.008 | 0.397 | 24.650 | < 0.001 | 0.175 | 0.205 |
| Age^2^ | -0.004 | < 0.001 | -0.217 | -13.509 | < 0.001 | -0.005 | -0.004 |
| Gender | 1.094 | 0.066 | 0.157 | 16.535 | < 0.001 | 0.964 | 1.223 |
| Note. *F*(3,10211) = 320.732, *p* < .001. *R*^2^= .086 | | | | |  |  |  |
|  |  |  |  |  |  |  |  |
| UK | *b* | *SE* | *β* | *t* | *p* | 95% LB | 95% UB |
| Age | 0.180 | 0.005 | 0.350 | 33.107 | < 0.001 | 0.169 | 0.191 |
| Age^2^ | -0.001 | < 0.001 | -0.038 | -3.596 | < 0.001 | -0.001 | < 0.001 |
| Gender | 1.026 | 0.043 | 0.149 | 24.014 | < 0.001 | 0.942 | 1.109 |
| Note. *F*(3,23370) = 1017.27, *p* < .001. *R*^2^= .116 | | | | |  |  |  |
|  |  |  |  |  |  |  |  |
| USA | *b* | *SE* | *β* | *t* | *p* | 95% LB | 95% UB |
| Age | 0.189 | 0.002 | 0.310 | 115.198 | < 0.001 | 0.186 | 0.193 |
| Age^2^ | -0.002 | < 0.001 | -0.060 | -22.309 | < 0.001 | -0.002 | -0.002 |
| Gender | 0.657 | 0.014 | 0.083 | 47.665 | < 0.001 | 0.630 | 0.684 |
| Note. *F*(3,307466) = 8556.864, *p* < .001. *R*^2^= .077 | | | | |  |  |  |

| Supplementary Table 8. Results for middle-age to older adulthood transition | | | | | | |  |
| --- | --- | --- | --- | --- | --- | --- | --- |
| Australia | *b* | *SE* | *β* | *t* | *p* | 95% LB | 95% UB |
| Age | 0.368 | 0.009 | 0.529 | 40.980 | < 0.001 | 0.350 | 0.385 |
| Age^2^ | -0.005 | < 0.001 | -0.151 | -11.722 | < 0.001 | -0.005 | -0.004 |
| Gender | 1.372 | 0.080 | 0.138 | 17.100 | < 0.001 | 1.214 | 1.529 |
| Note. *F*(3,12800) = 953.659, *p* < .001. *R*^2^= .183 | | | | |  |  |  |
|  |  |  |  |  |  |  |  |
| Belgium | *b* | *SE* | *β* | *t* | *p* | 95% LB | 95% UB |
| Age | 0.093 | 0.017 | 0.207 | 5.346 | < 0.001 | 0.059 | 0.127 |
| Age^2^ | < 0.001 | 0.001 | -0.001 | -0.039 | 0.969 | -0.001 | 0.001 |
| Gender | 0.928 | 0.144 | 0.141 | 6.453 | < 0.001 | 0.646 | 1.21 |
| Note. *F*(3,2069) = 37.305, *p* < .001. *R*^2^= .051 | | | | |  |  |  |
|  |  |  |  |  |  |  |  |
| Brazil | *b* | *SE* | *β* | *t* | *p* | 95% LB | 95% UB |
| Age | 0.148 | 0.013 | 0.223 | 11.605 | < 0.001 | 0.123 | 0.174 |
| Age^2^ | -0.001 | 0.001 | -0.039 | -2.018 | 0.044 | -0.003 | 0.000 |
| Gender | 0.898 | 0.096 | 0.124 | 9.401 | < 0.001 | 0.711 | 1.085 |
| Note. *F*(3,5435) = 99.155, *p* < .001. *R*^2^= .052 | | | | |  |  |  |
|  |  |  |  |  |  |  |  |
| Canada | *b* | *SE* | *β* | *t* | *p* | 95% LB | 95% UB |
| Age | 0.330 | 0.009 | 0.445 | 36.474 | < 0.001 | 0.313 | 0.348 |
| Age^2^ | -0.004 | < 0.001 | -0.126 | -10.358 | < 0.001 | -0.005 | -0.003 |
| Gender | 1.458 | 0.079 | 0.138 | 18.551 | < 0.001 | 1.304 | 1.612 |
| Note. *F*(3,15445) = 846.436, *p* < .001. *R*^2^= .141 | | | | |  |  |  |
|  |  |  |  |  |  |  |  |
| China | *b* | *SE* | *β* | *t* | *p* | 95% LB | 95% UB |
| Age | 0.207 | 0.015 | 0.176 | 14.134 | < 0.001 | 0.178 | 0.236 |
| Age^2^ | -0.002 | 0.001 | -0.024 | -1.966 | 0.049 | -0.005 | 0.000 |
| Gender | 0.239 | 0.091 | 0.031 | 2.629 | 0.009 | 0.061 | 0.418 |
| Note. *F*(3,7282) = 70.92, *p* < .001. *R*^2^ = .028 | | | | | | | |
|  |  |  |  |  |  |  |  |
| France | *b* | *SE* | *β* | *t* | *p* | 95% LB | 95% UB |
| Age | 0.342 | 0.011 | 0.466 | 32.397 | < 0.001 | 0.321 | 0.362 |
| Age^2^ | -0.006 | < 0.001 | -0.184 | -12.793 | < 0.001 | -0.007 | -0.005 |
| Gender | 1.293 | 0.086 | 0.131 | 15.117 | < 0.001 | 1.125 | 1.461 |
| Note. *F*(3,11664) = 569.052, *p* < .001. *R*^2^= 0.128 | | | | |  |  |  |
|  |  |  |  |  |  |  |  |
| Germany | *b* | *SE* | *β* | *t* | *p* | 95% LB | 95% UB |
| Age | 0.257 | 0.009 | 0.384 | 28.641 | < 0.001 | 0.240 | 0.275 |
| Age^2^ | -0.003 | < 0.001 | -0.101 | -7.556 | < 0.001 | -0.004 | -0.002 |
| Gender | 1.363 | 0.069 | 0.164 | 19.667 | < 0.001 | 1.227 | 1.499 |
| Note. *F*(3,12941) = 546.139, *p* < .001. *R*^2^= .112 | | | | |  |  |  |
|  |  |  |  |  |  |  |  |
| Korea | *b* | *SE* | *β* | *t* | *p* | 95% LB | 95% UB |
| Age | 0.141 | 0.016 | 0.146 | 8.901 | < 0.001 | 0.110 | 0.172 |
| Age^2^ | -0.003 | 0.001 | -0.045 | -2.818 | 0.005 | -0.005 | -0.001 |
| Gender | 0.436 | 0.106 | 0.054 | 4.125 | < 0.001 | 0.229 | 0.643 |
| Note. *F*(3,6161) = 30.714, *p* < .001. *R*^2^= .015 | | | | |  |  |  |
|  |  |  |  |  |  |  |  |
| Netherlands | *b* | *SE* | *β* | *t* | *p* | 95% LB | 95% UB |
| Age | 0.178 | 0.013 | 0.346 | 13.828 | < 0.001 | 0.153 | 0.203 |
| Age^2^ | -0.002 | 0.001 | -0.110 | -4.391 | < 0.001 | -0.003 | -0.001 |
| Gender | 0.573 | 0.106 | 0.075 | 5.411 | < 0.001 | 0.365 | 0.78 |
| Note. *F*(3,4981) =121.372, *p* < .001. *R*^2^=.068 | | | | |  |  |  |
|  |  |  |  |  |  |  |  |
| Spain | *b* | *SE* | *β* | *t* | *p* | 95% LB | 95% UB |
| Age | 0.271 | 0.015 | 0.370 | 18.618 | < 0.001 | 0.243 | 0.300 |
| Age^2^ | -0.002 | 0.001 | -0.073 | -3.686 | < 0.001 | -0.003 | -0.001 |
| Gender | 1.481 | 0.110 | 0.142 | 13.467 | < 0.001 | 1.265 | 1.696 |
| Note. *F*(3,8224) = 324.925, *p* < .001. *R*^2^= .106 | | | | |  |  |  |
|  |  |  |  |  |  |  |  |
| Sweden | *b* | *SE* | *β* | *t* | *p* | 95% LB | 95% UB |
| Age | 0.314 | 0.009 | 0.542 | 35.561 | < 0.001 | 0.297 | 0.332 |
| Age^2^ | -0.005 | < 0.001 | -0.211 | -13.862 | < 0.001 | -0.006 | -0.004 |
| Gender | 1.568 | 0.076 | 0.185 | 20.669 | < 0.001 | 1.420 | 1.717 |
| Note. *F*(3,10233) = 752.477, *p* < .001. *R*^2^= .181 | | | | |  |  |  |
|  |  |  |  |  |  |  |  |
| UK | *b* | *SE* | *β* | *t* | *p* | 95% LB | 95% UB |
| Age | 0.252 | 0.006 | 0.408 | 38.864 | < 0.001 | 0.240 | 0.265 |
| Age^2^ | -0.002 | < 0.001 | -0.091 | -8.732 | < 0.001 | -0.003 | -0.002 |
| Gender | 1.365 | 0.051 | 0.164 | 26.771 | < 0.001 | 1.265 | 1.465 |
| Note. *F*(3,23379) = 1165.347, *p* < .001. *R*^2^= .130 | | | | |  |  |  |
|  |  |  |  |  |  |  |  |
| USA | *b* | *SE* | *β* | *t* | *p* | 95% LB | 95% UB |
| Age | 0.280 | 0.002 | 0.344 | 129.653 | < 0.001 | 0.276 | 0.285 |
| Age^2^ | -0.003 | < 0.001 | -0.068 | -25.779 | < 0.001 | -0.003 | -0.002 |
| Gender | 1.227 | 0.018 | 0.116 | 67.671 | < 0.001 | 1.192 | 1.263 |
| Note. *F*(3,307935) = 11294.627, *p* < .001. *R*^2^= .099 | | | | |  |  |  |

Supplementary Figure 1. Perceived age effects across 13 countries


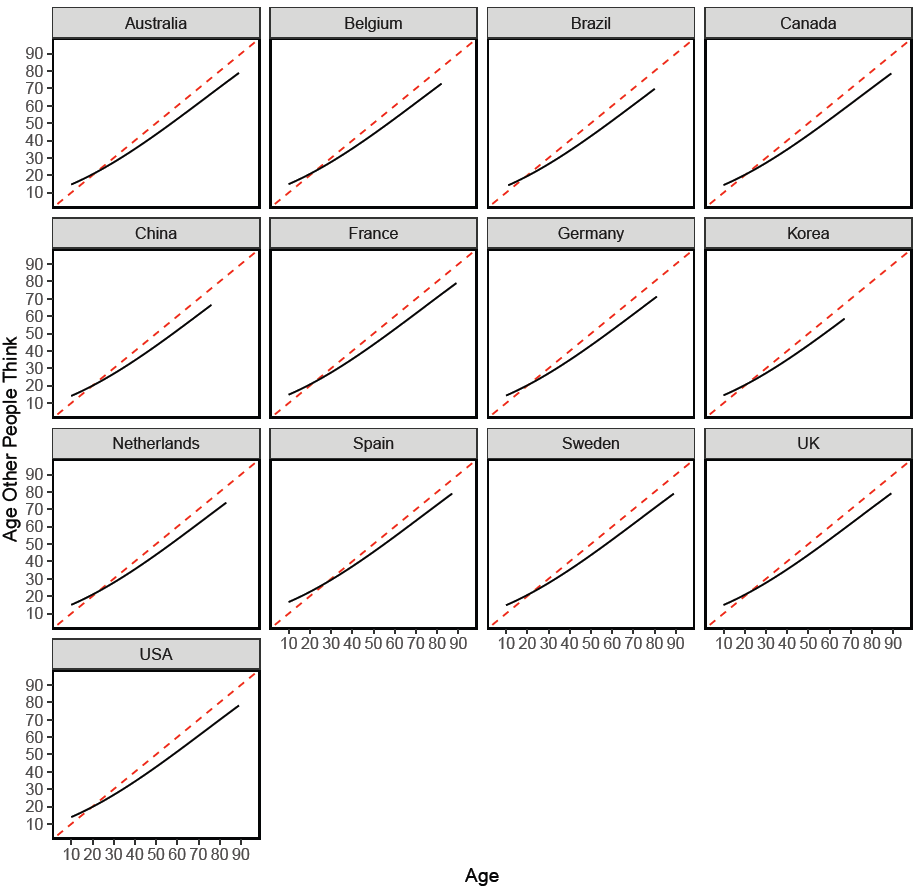


Supplementary Figure 2. Ideal/chosen age effects across 13 countries


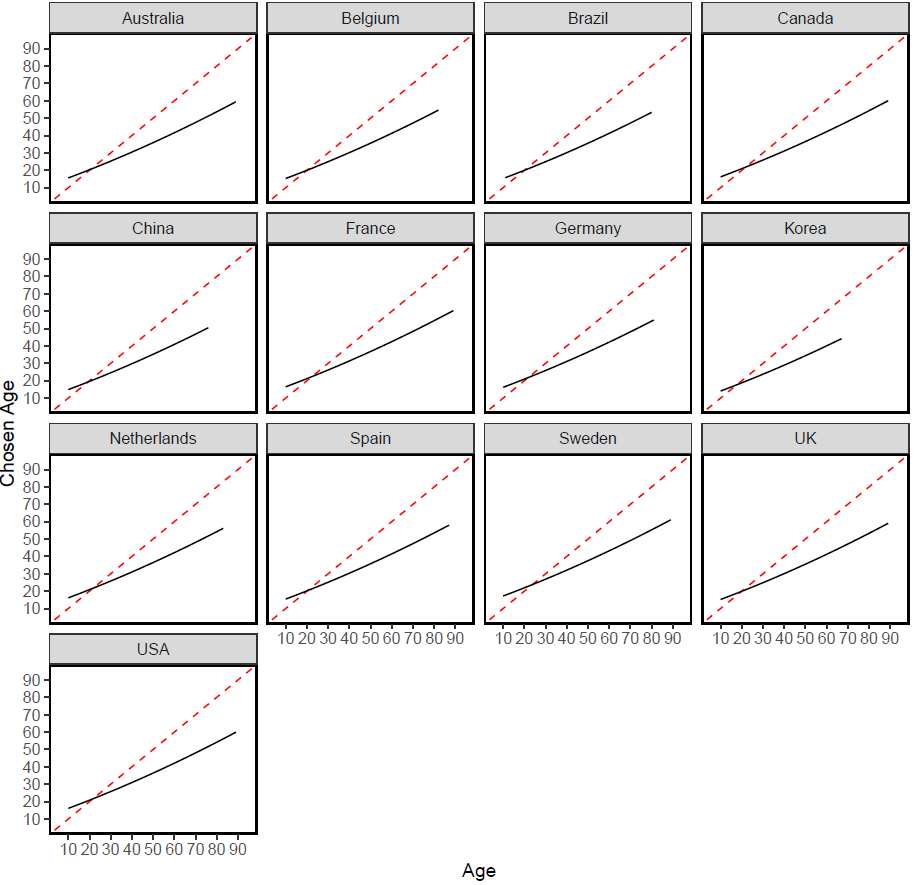


Supplementary Figure 3. Ideal age to live until across 13 countries

**
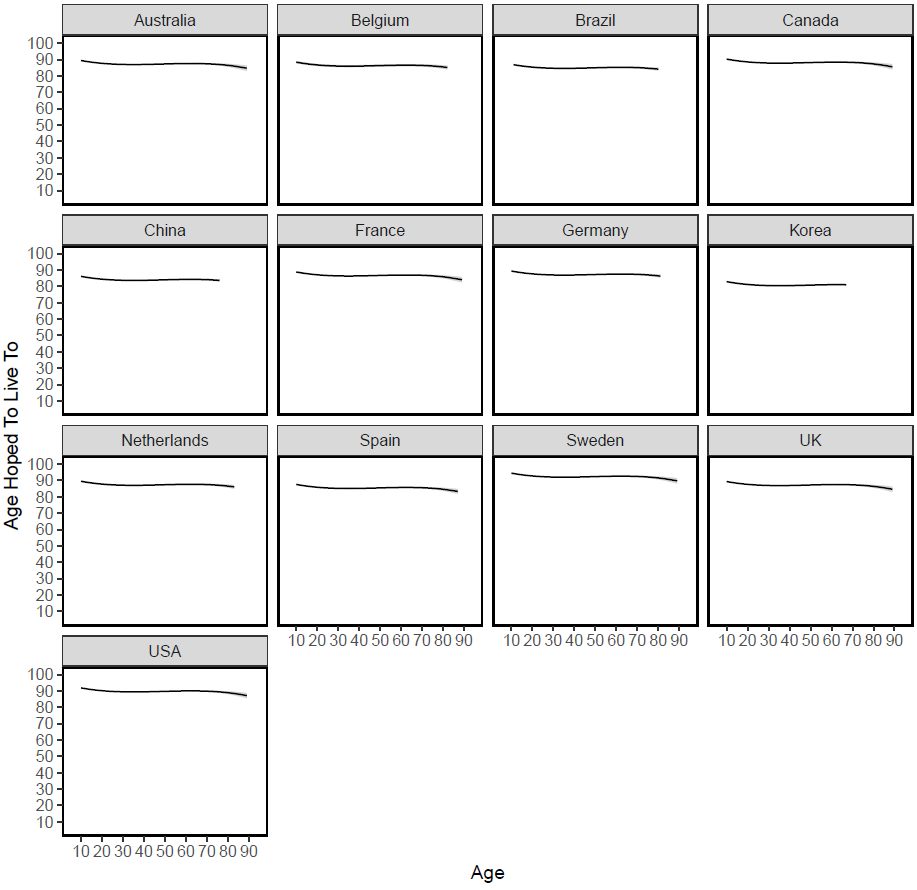
**

Supplementary Figure 4. Perceived developmental transitions across 13 countries broken down by country (zoomed out)


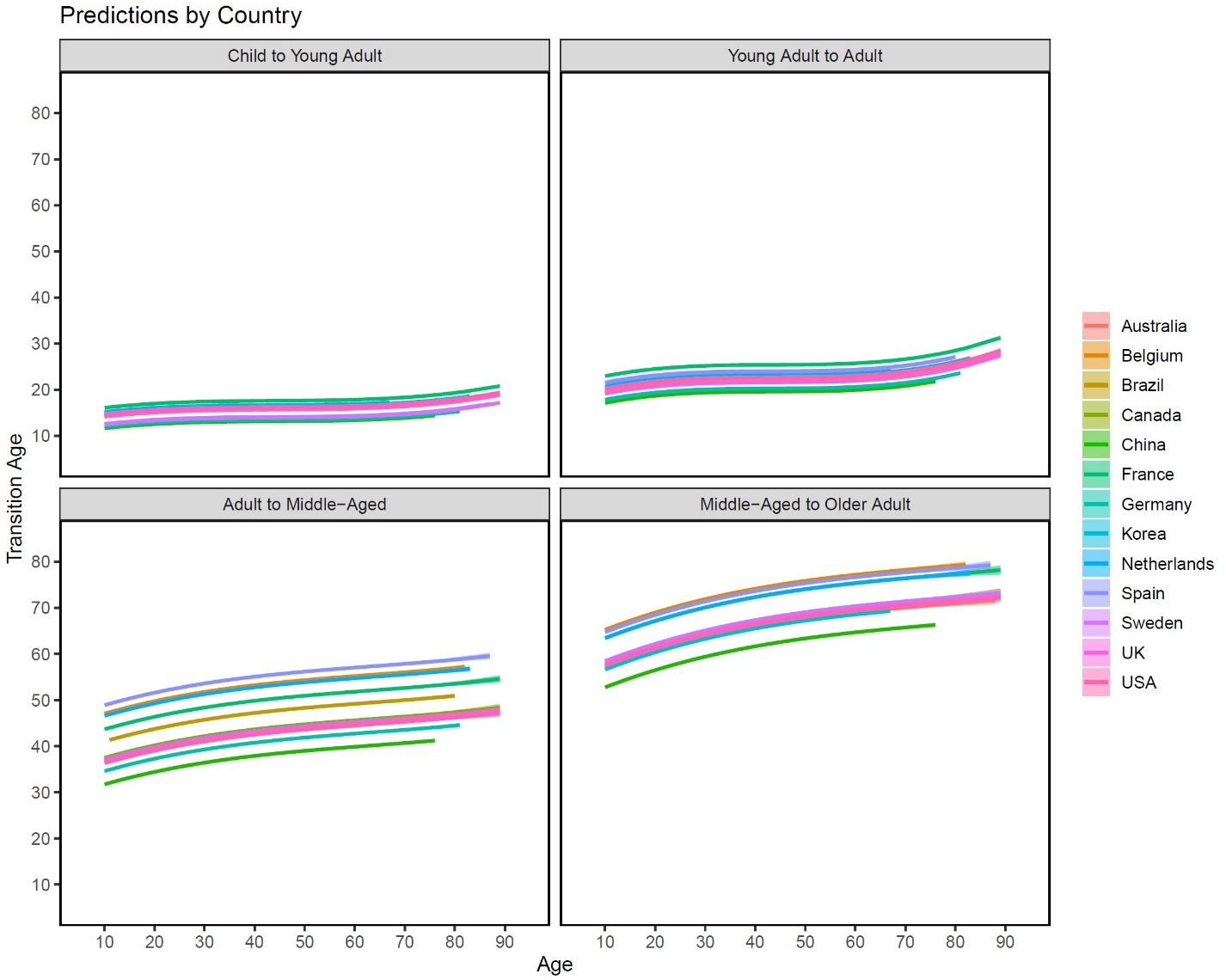

Supplement: Supplementary material [file NIHMS2106334-supplement-Supplementary_material.docx]
